# Religion and Fertility: A Longitudinal Register Study Examining Differences by Sex, Parity, Partner’s Religion, and Religious Conversion in Finland

**DOI:** 10.1007/s10680-023-09693-0

**Published:** 2024-02-19

**Authors:** Martin Kolk, Jan Saarela

**Affiliations:** 1 Demography Unit, Department of Sociology, Stockholm University, Stockholm, 10691 Sweden; 2 DemSwed, Åbo Akademi, Vasa, Finland; 3 Institute for Futures Studies, Stockholm, Sweden

**Keywords:** Religion, Fertility, Demography, Homogamy, Secularization, Finland

## Abstract

We use longitudinal data on religious affiliation in Finland to examine childbearing behavior. All analyses are based on detailed fertility information from the Finnish national register of each person’s religious denomination for men and women born in 1956–1975. We identify higher fertility according to parity among members of the Evangelical Lutheran state church and other Protestant churches, and lower fertility among individuals with no religious affiliation. Most other religious groups—Orthodox Christians, Jews, Muslims, and adherents of Eastern religions—have intermediate levels of fertility. We also find that religious converts, that is, those observed with more than one religious denomination over their life course, typically are similar to the non-converts of the group they convert to, though with more distinct deviations from the Finnish population. Women show larger differences by religious affiliation than men. We find the largest differences across religions when we examine the proportion of childless men and women. Overall, differences between religious groups are rather modest, and childbearing patterns are quite similar. Our results provide, to our knowledge, the first examination of religion and fertility using national-level longitudinal data.

**Supplementary Information:**

The online version contains supplementary material available at 10.1007/s10680-023-09693-0.

## Introduction

Religion is a central dimension for many explanations of demographic change. It has been an important issue in theories of family change in high-income countries since the 1960s, as increasing individualization and secularization have been linked to lower fertility (Lesthaeghe, 1995; Thornton, 2005). A decline in organized religion and religious worldviews has been seen as a part of the general modernization process.

Many demographic studies of religion have been concerned with secularization, the intensity of religious beliefs, and how they affect childbearing preferences and outcomes (Frejka & Westoff, 2008; McQuillan, 2004; Mosher et al., 1992). In this study, we take a different perspective from much previous research, which has linked fertility to religiosity and religious practices. We focus instead on differences in childbearing across denominations and religious groups. In some contexts, such as the USA, there exists much research on fertility differentials between Christian denominations, with a particular focus on differences between mainline Protestant denominations, Catholics, Evangelical Protestants, and the unaffiliated (Frejka & Westoff, 2008; Hackett, 2008). A smaller literature has studied these issues from a global perspective, sometimes using data on religion and fertility to estimate the future population composition of religious groups (Hackett et al., 2015). Fertility differences by religion are, together with trends in religious change, the primary determinants of the future composition of the religious landscape in the world (Stonawski et al., 2015).

We expand on the topic by examining differences by religious denomination in the highly secular Nordic context, for which there is comparatively little previous research on the interrelation between religion and fertility. We do this by applying data from Finland, which maintains longitudinal population registers of not only demographic variables but also each person’s religious denomination. This allows us to study differences by minority religious affiliation, even though Finland was, in terms of religion, a rather homogenous country during the 20th century.

By using administrative register data instead of surveys, we make several contributions to the study of religion and fertility. First, we use national-level population data with highly accurate measurements of both fertility and religion, examining Finnish men and women born between 1956 and 1975, who have been resident in Finland after age 15. This allows us to examine quite small denominations, and aspects such as parity differences. We also do this by sex, examining fertility separately for men and women, thus taking advantage of the availability of reliable fertility data for men. Some of the Christian denominations we study, such as Catholics, are small within the Finnish religious landscape, but more frequent in other high-income countries. For some, like Jehovah’s Witnesses, representative data on fertility have been lacking in any country or context. Some are relatively rare in the cohorts in our data but constitute a rapidly growing share of the European religious landscape due to international migration, such as Muslims and Hindus. The growing importance of non-European religions is a common theme in research on the sociology of religion in Europe (Guveli & Platt, 2023).

Second, we use high-quality longitudinal register data with yearly information on membership by religious denomination. This allows us to examine fertility differences between converts (those observed with at least two religions over their life course) and non-converts (those observed with only one religion). The fertility of converts, has to our knowledge, seldom been studied in research on religion and fertility. This is particularly important as converts, which here includes also those who go from a religious background to no denomination or vice versa, are the agents of a transformation of the religious landscape in a country.

Third, we also incorporate partners’ religions, in order to distinguish how the interaction between one’s own and one’s partner’s religion affects fertility. By focusing on processes of religious homogamy and exogamy, we highlight how fertility in nearly all cases is a negotiated and gendered experience between two partners. We can thus examine both how homogamy and exogamy in itself can affect fertility and if this differs across denominations, and in addition, the extent to which religious homogamy among different denominations affects fertility.

## Previous Research on Religion and Fertility

For over a century, a large body of research has examined the link between religion and fertility. In prominent explanations of the demographic transition—and the associated transformative fall in fertility—cultural change, intrinsically linked to secularization, has been highlighted as of fundamental importance for falling fertility (Lesthaeghe (1983). Already in Notestein’s (1945) formulation of the demographic transition theory, the weakening of religious doctrines was an important explanation for fertility decline, as it was argued that religion had previously maintained fertility at a high level.

In light of religion’s importance as a mechanism behind fertility, much empirical work has linked the two concepts. A common finding is that the intensity of religious beliefs and practices is a strong positive determinant of fertility (Berghammer, 2012; Frejka & Westoff, 2008; Hackett, 2008; Mosher et al., 1992; Zhang, 2008). It has also been argued that religious practices are more important than belief and affiliation (Philipov & Berghammer, 2007). Some research suggests that differences in fertility across religious groups have decreased in importance over time, but that the salience of individual religiosity within groups has remained an important determinant (Kaufmann, 2010; Zhang, 2008). While the highest fertility is found among the most religious, the lowest fertility is often found among the non-affiliated, and explicitly atheistic individuals have indeed the lowest fertility (Frejka & Westoff, 2008; Hackett, 2008). The importance of non-affiliation is particularly relevant in the Finnish context, as the two largest religious groups consist of members of the Finnish state church and those who have actively decided to leave the state church and are unaffiliated.

Different suggestions have been given for why religious people often have more children (Philipov & Berghammer, 2007). Most proximate to childbearing, some religions are skeptical toward contraception and abortion for theological reasons. In modernization theories, religion is often seen as a part of an earlier more traditional way of life, with a larger focus on family, traditional gender roles, and conservation of traditional lifestyle, in contrast to life paths that seek individual self-actualization outside a conventional traditional family involving childbearing (cf. Lesthaeghe, 1983; Thornton, 2005). It is noteworthy, that Christianity for long theologically had an adverse relationship with family and kinship (Goody, 1983), though this began to change with the Enlightenment, when churches more clearly began to identify themselves with traditional family values. How different denominations focus on traditional family life, ideas about contraception, and endogamy differs greatly across denominations, and different denominations in Finland also differ greatly in the extent of religious practice. One would therefore expect differences in fertility across denominations.

Since our study is focused on differences by religious denomination, we will begin by discussing previous research documenting trends in average fertility across religious denominations, and focus less on the larger literature focusing on religiosity and fertility. US studies have documented higher fertility among Evangelical Protestants and black Protestant churches (Hackett, 2008), a difference also found elsewhere (Dilmaghani, 2019; McKinnon et al., 2008). Mainline Protestant churches have lower fertility than the population average, with Catholics in between. Recent studies suggest that, while for most of the second half of the twentieth century, fertility was very high among Evangelical Christians, these groups have also started to see substantial fertility declines since the 1990s (Perry & Schleifer, 2019).

Little is known about fertility among members of what, in a Nordic context, are known as free churches, which are primarily associated with Christian revivals during the nineteenth and early twentieth centuries and, later, congregations inspired by American charismatic Christianity (Iversen, 2006). The name free churches is due to their origin outside the National Lutheran church, and this label is also how their ecumenical tent organization chooses to refer to themselves in English. These groups amount to a substantial share of committed and practicing Christians in the Nordic context (Iversen, 2006). An exception is various pietist and other religious revival organizations working within the Lutheran state church, such as the Laestadians, who are committed and practicing Christians with high fertility, but belong to the Evangelical Lutheran state church (Finnäs, 1991). The Laestadians have put particular emphasis on children as a gift of God, and often did not use any contraceptives (Finnäs, 1991).

When we focus on differences across denominations, we note that Protestant denominations—both in the US and Nordic contexts—are largely distinguished by the intensity of the religiosity of their adherents (Hackett, 2008). Membership is therefore often substantially self-sorted based on the religious preferences of the members. Thus, the strength of religiosity is likely an important determinant distinguishing different Protestant denominations.

Fertility among Catholics has been central in much historical traditional theorizing on religion and demographic change in both the USA and Europe, focused on their relativly higher fertility than other groups. Recent studies find that Catholics have lower fertility than Protestants in many European countries, with small differences in the USA (Frejka & Westoff, 2008; Mosher et al., 1992). There are still some contexts such as the UK, where Catholics have higher fertility (Peri-Rotem, 2016). These trends mirror fertility at the cross-national level, where Catholic European countries used to have high fertility and are now instead among lowest low-fertility countries (Berman et al., 2018). In some European contexts such as Spain, researchers find quite small differences by religion (Adsera, 2006; Mogi et al., 2022).

A consistent finding across most contexts is lower fertility among the non-religiously affiliated (Frejka & Westoff, 2008; Hackett et al., 2015; Lehrer, 1996). It seems that most aspects of non-religiosity, such as non-attendance, lack of beliefs, non-affiliation, and expressed atheism, are in most contexts linked to lower fertility (Frejka & Westoff, 2008; Hackett, 2008).

Jewish fertility is consistently low in the USA and also, most likely, in Western Europe (Hackett, 2008; Mott & Abma, 1992). This is in contrast to the high-income context of Israel, where fertility remains at a relatively high level, particularly among more religious Jews, but also among secular ones. Historically, Jews in Eastern Europe have had relatively high fertility, though fertility among Jews living in Western Europe was (Livi-Bacci, 1986), and may still be, lower than that of the majority population.

Muslim fertility in Europe is often described as high, though less is known about fertility in the Nordic countries. It seems that many recent immigrant groups from Muslim countries have fertility levels that are a bit higher in the first generation (Stonawski et al., 2016; Westoff & Frejka, 2007), and comparable or lower in the second generation compared to the majority population (Andersson et al., 2017). In Western and Central Europe, Muslims more consistently have higher fertility than the majority population, though the increase is rather moderate (Westoff & Frejka, 2007). In Southeastern Europe, where Islam has a long history, fertility is higher among Muslims than among other groups (Stonawski et al., 2016; Westoff & Frejka, 2007).

Orthodox Christian fertility in the USA is below the population average (Hackett, 2008), while little is known about Orthodox fertility in Europe, though immigrant groups from Orthodox European countries that live in Central and Western Europe often show the same low fertility observed in their countries of origin (Andersson et al., 2017).

Less is known about smaller and rapidly growing Christian religious affiliations in Europe, such as the Church of Latter Day Saints (hereafter Mormons) and Jehovah’s Witnesses, though in the USA, high fertility among Mormons is well documented (Hackett, 2008; Lehrer, 1996). Little is known about fertility among the Jehovah’s Witnesses, but some evidence suggests it is higher than the population average (Stark & Iannaccone, 1997). In contrast to the Abrahamic religions, there is little evidence that Buddhism is positively related to fertility (Skirbekk et al., 2015). Not much is known about the link between religiosity and other East Asian religions, particularly in a European context. Overall, research on religion and fertility in Europe has often been limited by surveys with few respondents, where researchers have only had access to surveys which have made it hard to study minority religious groups.

In contrast to the USA and continental Europe, there has been little research on the link between childbearing and religion in the Nordic countries. One exception is a study by Finnäs (1991) of the high fertility of the Laestadians in a local Finnish setting. Researchers in the Nordic countries have otherwise examined the fertility transition in the nineteeth and early twentieth centuries and (implicitly) related it, in different ways, to religiosity (Junkka & Edvinsson, 2016; Larsson, 1984; Sundt, 1857[1993]). Carlsson (2022) examined fertility in Sweden using survey data and found higher fertility among native free church members and slightly lower fertility among the non-affiliated than affiliates of the state church. Among migrants, he found elevated fertility among both Muslims and Christians relative to non-religious individuals.

With few exceptions, nearly all the findings we report above refer to female fertility and female religiosity, while we know substantially less about male fertility behavior, and particularly its interrelation with religion. We note that there are debates on the gendered nature of religiosity and spirituality, and on how they differ across religions. Broadly speaking, Christian religions are often characterized by more female adherents and stronger religiosity among women, while for some other religions, such as Islam, religiosity is, at least publicly, more important for men (Francis & Penny, 2013). Most previous research on religion and fertility has focused on women and little has focused on male fertility or fertility following intermarriage (though see, Adsera, 2006; Lehrer, 1996), which we examine here. Lehrer (1996) studied conversions in the USA and found that women, who marry out from their religion of origin, have depressed fertility among Mormons and Catholics. With a few exceptions, previous studies discussed above have been limited in their data material, and not able to either study converts/non-converts or how homogamy in partner religion affects childbearing by denomination.

Our study is broad and descriptive in nature. The results will therefore reflect many compositional and selection aspects of the minority religions we analyze. In many cases, the contexts and group compositions likely differ substantially from contexts where the religious group is a majority denomination in the society. To examine these issues, we will control for socioeconomic conditions in our population. They reflect differences in observable characteristics and not the social and cultural context of belonging to a religion, which is an important feature in many other parts of the world. In our population, individuals from religions of non-European/Abrahamic origin are mostly born in Finland and are thus not necessarily representative of the more recently arrived large immigrant populations in the rest of Europe. However, this does not affect the internal validity of our study.

## Demography and Religion in Finland

The Nordic region is often considered a “forerunner” of many family demographic trends, such as the increase in cohabitation and divorce, and the decreases and delays in marriage and marital childbearing (Lesthaeghe, 2010). Finland, like its Nordic neighbors, has often been used to exemplify countries in the vanguard of the so-called second demographic transition, where falling fertility is linked to secularization (Lesthaeghe, 2010; Van de Kaa, 1987). Lately, Finland, like the other Nordic countries, has experienced a relatively rapid fertility decline (Comolli et al., 2021). At the same time, there was a rise in individualistic values related to family formation, and legal and social policy changes promoted a more gender-equal and individualized family system (Therborn, 2004).

Finland has undergone rapid secularization, and is often included among the most secular nations in Europe (Voas & Doebler, 2011). Religious affiliation has been falling for several decades, from over 90% in the early 1970s to less than two thirds of the population (Xia et al., 2023). The country has two national churches, where the Lutheran church has a similar background to the state churches in Sweden, Norway, and Denmark. The two state churches are the Evangelical Lutheran Church of Finland (around 2/3 of the population) and the Orthodox Church of Finland (1%). Due to the overlap between the state church and government, and the historical role of the church in population administration, Finland has collected data on religious denominations for a long period. Government registration, where individuals are registered as part of a religious denomination (including no denomination), is regulated by law. Unlike in other Nordic countries, these data also include smaller denominations.

While church membership is still high in Finland, religious practice among most members of the state church is low, and for many, the church fulfills more of a cultural than a spiritual role in people’s lives (Iversen, 2006). To interpret patterns within this large group, it is thus useful to relate this population to the large groups of “nominally” affiliated individuals in other European countries (Peri-Rotem, 2016). Within this group, there are practicing members who regularly attend church, but they remain a minority of all state church members. In 2020, 30% of the population in Finland had no religious affiliation, a share that increased steadily over our study period (Xia et al., 2023). In supplemental text S1, we use survey data from the European Social Survey to explore how survey respondents describe their religious beliefs and practices. We find that self-rated religiosity is high in Finland, also among members of the state church. In contrast, religious attendance is low, with individual religious practices such as praying having an intermediate position. The difference between those identifying with no religion and the state church is very large, indicating that self-identifying with the state church has clear sociological and spiritual meaning and is socially salient.

Unlike in the other Nordic countries, much of the activity during the religious revivals of the nineteeth and early twentieth centuries was in Finland integrated into the Evangelical Lutheran state church (Iversen, 2006). Many, but not all, of these congregations are therefore formally integrated into the national church, and at a higher rate than in the other Nordic countries. The Laestadians are one example (Finnäs, 1991) and they are, in our data, consequently counted as members of the state church. In supplemental text S1, we show that members of free churches and other smaller Christian denominations have much higher levels of religious practice and religiosity than members of the state church.

Under Russian rule, a small community of Tartars settled in Finland, organizing their own mosques, and remaining distinct from more recent Muslims arriving in Finland. Finland also has a small but well-integrated Jewish community since the nineteenth century. The minority native Sami population in Northern Finland has, and to some extent continues to practice a religion based on Shamanism and Animism, but this is not organized into a religious structure/organization that is included in national data (and is thus not reflected in our data). Over time, many Sami have become members of the state church and the Laestadian community.

Finland is a bilingual state with a minority of Swedish speakers and a majority of Finnish speakers. There are more non-affiliated individuals in the Finnish-speaking majority population (Xia et al., 2023). Protestant churches that are not part of the state church have an overrepresentation of Swedish speakers, and the Jewish community in Finland is majority Swedish-speaking (Xia et al., 2023).

## Data and Methods

Our data are based on total population counts of all individuals who lived in Finland at any time between 1970 and 2020. Through parent–child linkages, we could create accurate childbearing histories for both men and women. Linkages were accessible due to personal identity numbers, which allow seamless and accurate matching. Uniquely for countries with longitudinal national population registers, Finnish population registers keep track of religious data, including for members of non-state church denominations. This information, which was collected for every individual and every year 1971–2020, is integrated into the longitudinal population register and updated on a yearly basis.

Our raw data contain information on about 50 different denominations, which we aggregated into nine groups: (1) the Evangelical Lutheran state church, (2) no religion (without any affiliation), (3) Orthodox Christians (the Orthodox state church plus Orthodox denominations that are not part of it), (4) other Protestants (various Protestant churches independent of the state church), (5) other Christians (denominations such as Jehovah’s Witnesses and the Latter Day Saints church), (6) Catholicism, (7) Islam, (8) Eastern (various denominations, such as Buddhists, Bahai, and Hindus), and (9) Judaism. We ordered them in our data by relative size. For our summary table, we additionally break down the (3) Orthodox, (4) other Protestant, and (5) other Christian groups into smaller denominations within these larger groupings, while all other results are based on the grouping above.

For our main analyses, we restricted the data to persons born in 1956–1975. We could thereby observe each individual’s complete childbearing history up to age 45 and religious domination at age 15 (before childbearing starts) and age 45. To ensure that records on childbearing were complete, we further restricted the study population to individuals who had consistently lived in Finland in ages 15–45. Those who emigrated and/or return migrated between ages 15 and 45 were thus excluded, and so were also those who died before age 45. Our population consisted of 629,038 women and 650,044 men.

Since we studied the population born before 1976 that never migrated after age 15, and conditional on residence in Finland at age 15, individuals with an international migration history were mostly excluded from our analysis. Immigration to Finland was very modest before the 1990s. The number of members in some religious denominations according to our classification—Catholic, Muslim, Eastern, and Jewish—are therefore small, and contain only a few hundred individuals. We thus performed parallel analyses with the same criteria described above, but which also included the cohorts born in 1938–1955. This study population was approximately twice as large. These additional results are broadly similar to the others reported here and are available upon request. For these older cohorts, fertility is nevertheless somewhat undercounted because parent–child linkages in 1970 were conditional on co-residence and not on birth records, and religious denomination cannot be observed at age 15 before 1955.

In the results section, we present the mean number of children by religion at age 45 for women and men born in 1956–1975. For each sex, we have calculated the contribution by parity and displayed eventual childlessness by religious affiliation. The contribution by parity is calculated by breaking down both the numerator and denominator by parity (the same is done for partner’s religion). We compare persons who converted to each religious denomination to those who did not convert, where the first group considers anyone with two or more observed religions (including those moving to/from having no denomination), and the second group those that are observed with the same denomination across our data. When showing results for converts, we group them by their last observed religion. We have also calculated both the average fertility of each index individual and his or her first childbearing partner’s religious denomination. We separate between those with a partner of the same religion, those with a partner in the state church, those with a partner with no religion, and those with an exogamous marriage not in the last two groups. These calculations are done for each birth, and the partner of that birth, of an index individual. Poisson regressions were estimated to evaluate whether socioeconomic and demographic variables affected fertility by religion. In these models, we use information on education, mother tongue, marital status, and municipality, derived from Finnish register data and measured at age 45. These variables were introduced stepwise into the models, beginning with basic demographic controls, followed by sociodemographic background (education and mother tongue), followed by marital status to see if the fertility differences remained also among partnered individuals. We report average marginal effects based on Poisson regressions, which can be straightforwardly interpreted as differences in the mean number of children and easily compared to our descriptive results. The full regression output is available in Tables A1 and A3–A5 (supplementary tables S2). We attach all our aggregated data used to make our figures in supplemental file S3.

## Results

**Fig. 1:**
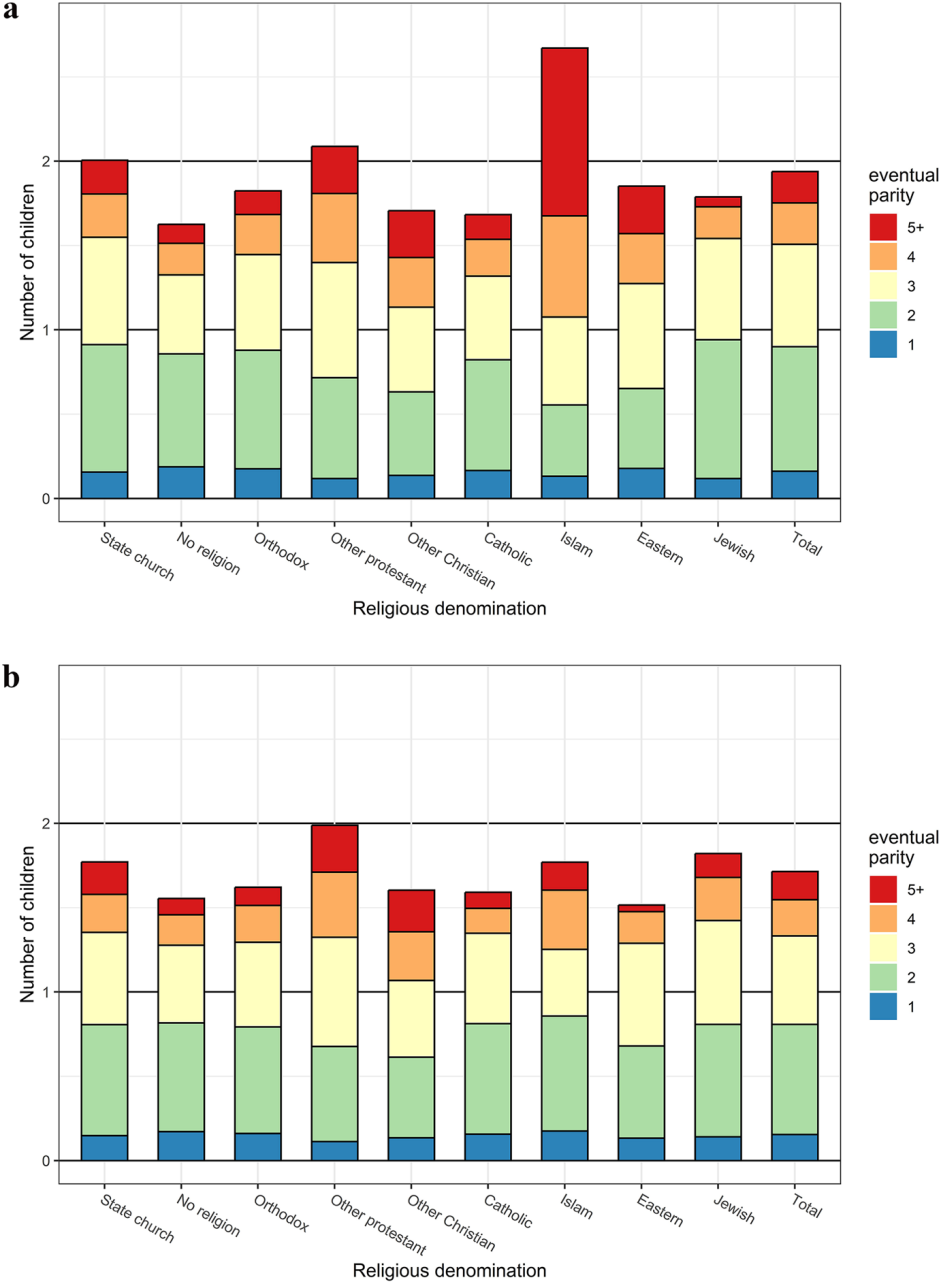
Mean number of children at age 45 by religious denomination, broken down by contribution of final parity to the mean, women (a, top panel) and men (b, bottom panel)

Table 1 presents the mean number of children at age 45 by religious denomination for women and men. Figure 1 shows the average fertility of men and women, and in addition shows how individuals with different eventual parity contribute to this average (the sub-areas of the stacked bars). Fertility differences across religious denominations are larger for women than for men. Among women, members of the state church and other Protestant churches have higher fertility than members of other denominations (except for those registered as Muslim) with, on average, 2.00 and 2.09 children, respectively (Table 1). Among men, members of the state church, other Protestants, and Jews have the highest fertility with, on average 1.77, 1.99, and 1.82 children, respectively. Both Orthodox and Catholic women and men have lower fertility than men and women of the state church. The group without religious affiliation has low fertility, or on average 1.63 children among women and 1.55 children among men. This secularization effect is thus larger among women than men, as the average number of children is 0.37 lower for women and 0.22 lower for men, compared to members of the state church. Other Protestants have, on average, both larger families and a higher proportion with very high parities than people in the general population (Fig. 1), and this differs little among denominations within this group (Table 1). The group with other Christians has fewer children than the average population, which is entirely due to low fertility among Jehovah’s Witnesses, while Mormons have large families (Table 1). Both women and men without a religious denomination have relatively small families, as do Orthodox and Catholic women and men. The same holds true for Jewish women and Eastern men. Women who belong to Islam and Eastern religions have a higher share of individuals with unusually high parities. The fertility of Jewish men is slightly higher than that of the total population.

**Table 1:** Mean number of children at age 45 by religious denomination, women and men

	Women			Men		
	Mean fertility	Group size		Mean fertility	Group size	
		*N*	%		*N*	%
**State church**	2.00	509,121	80.9	1.77	471,847	72.6
**No religion**	1.63	106,375	16.9	1.55	166,611	25.6
**Orthodox Christian**	1.82	6,049	1.0	1.62	5,099	0.8
*Orthodox state church*	1.82	6,000	1.0	1.62	5,024	0.8
*Other Orthodox*	1.69	49	0.0	1.75	75	0.0
**Other Protestant**	2.09	3,630	0.6	1.99	3,248	0.5
*The Evangelical Free Church of Finland*	2.10	2,006	0.3	2.02	1,730	0.3
*Baptists and Adventists*	2.07	700	0.1	1.94	590	0.1
*Methodists, Lutherans, and Anglicans*	2.01	275	0.0	1.73	374	0.1
**Other Christian**	1.71	2,934	0.5	1.60	2,399	0.4
*Jehova’s witnesses*	1.56	2,578	0.4	1.45	2,061	0.3
*LDS church (Mormons)*	2.77	349	0.1	2.50	336	0.1
**Catholic**	1.68	496	0.1	1.59	543	0.1
**Islam**	2.66	213	0.0	1.77	91	0.0
**Eastern**	1.85	135	0.0	1.52	128	0.0
**Jewish**	1.79	85	0.0	1.82	78	0.0
**Total**	1.94	629,038		1.71	650,044

**Fig. 2:**
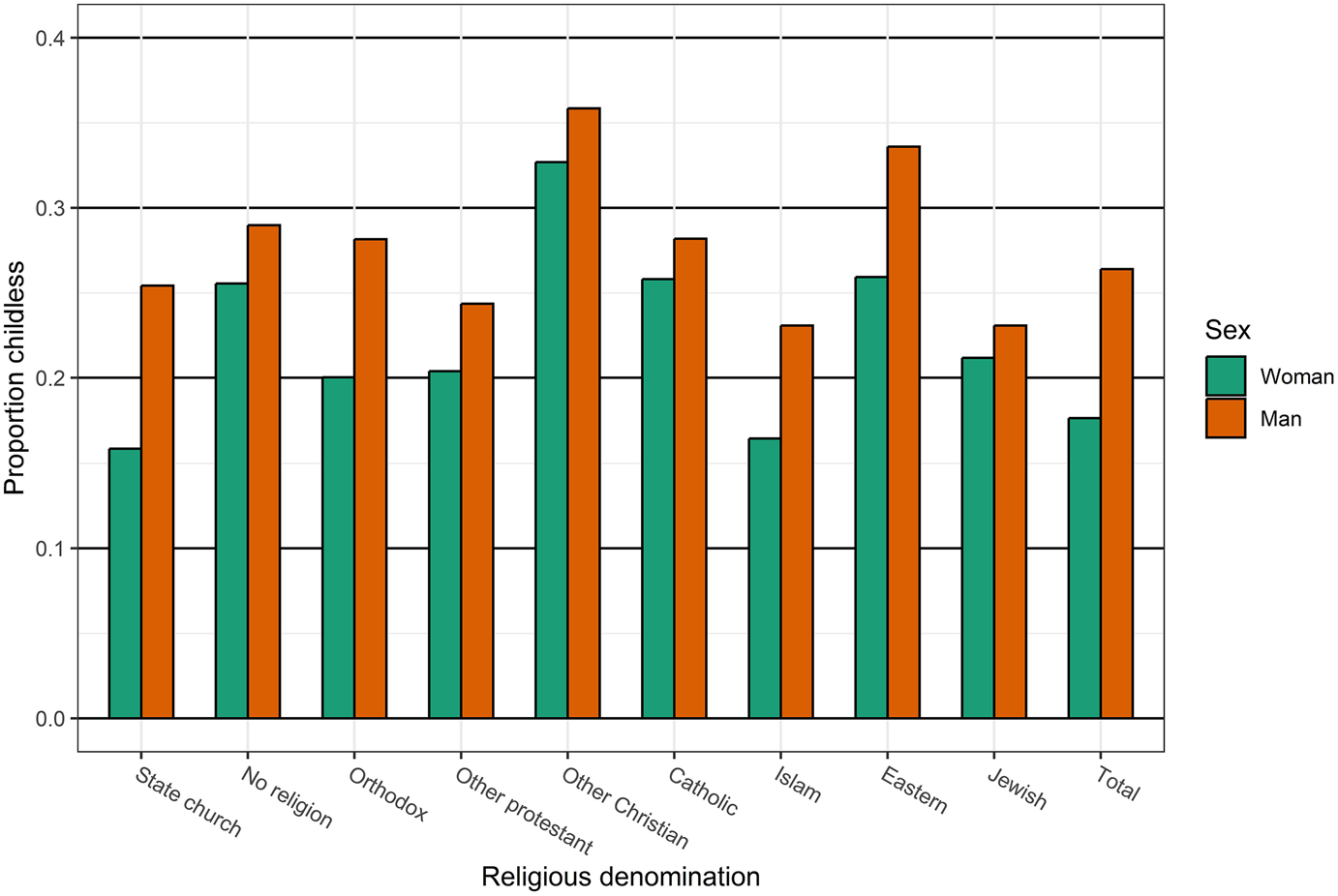
Proportion childless at age 45 by religious denomination, women and men

For childlessness, we see larger variation across religious denomination than for fertility in general. Figure 2 shows the proportion of women and men who are eventually childless at age 45 by religious denomination. As is typically found in high-income countries, childlessness is higher among men than women due to both sex differences in multi-partner fertility and the larger number of men in the population. The sex difference in childlessness is consistent across all denominations, but particularly marked among members of the state church, the Orthodox, Muslims, and Eastern denominations. Among the non-religious, the sex difference in childlessness is relatively small. The share of childless women is lowest among members of the state church and Islam, at just over 0.15. The highest share of childlessness among women is found among other Christians for every third individual, followed by the non-religious and Catholics, for every fourth individual. Among men, other Christians and Eastern denominations are associated with high levels of childlessness, or more than every third individual. Men without a religious denomination, the Orthodox, and Catholics are somewhat more likely to be childless than members of the state church, while other Protestants, Muslims, and Jews are less likely to be childless.

Birth cohort, mother tongue, and educational level have only a modest influence on the association between religious denomination and fertility. Table 2 displays the average marginal effects of religion on fertility for women and men, where the state church serves as the reference category. The estimates of Model 1 correspond to the differences in means as shown in Table 1 and Fig. 1. These estimates remain largely unaffected when we include year of birth, mother tongue, and educational attainment (Model 2). Fertility differences by religious denomination are generally reduced when municipality of residence is accounted for (Model 3), and even more so when marital status is added (Model 4). The difference between members of the state church and people with no religious denomination is then -0.25 children for men and -0.17 for women. For all other religious denominations—except Islam among women, Eastern religions among men, and other Christians for both men and women—the difference from the state church is at most 0.10 children, and not statistically significant. The elevated fertility of members of Islam is amplified when the municipality of residence is introduced, and the depreciated fertility of other Christians becomes larger when marital status is included. These patterns are reinforced when religious conversion is added in Model 5. Having changed religion between the ages of 15 and 45 thus underlies part of the fertility difference between members of the state church and the non-affiliated, and a minor part of the difference between members of the state church and the other religious denominations. Our findings for women and men are similar in this respect.

**Table 2:** Average marginal effects (with standard errors and p-values) of religious denomination at age 45 on the number of children at age 45, by sex

	Model 1			Model 2			Model 3			Model 4			Model 5		
	*d*y/*d*x	SE	*P* >|*z*|	*d*y/*d*x	SE	*P* >|*z*|	*d*y/*d*x	SE	*P* >|*z*|	*d*y/*d*x	SE	*P* >|*z*|	*d*y/*d*x	SE	*P* >|*z*|
*Women*															
State church	Ref			Ref			Ref			Ref			Ref		
No religion	− 0.38	0.00	0.00	− 0.38	0.00	0.00	− 0.30	0.00	0.00	− 0.25	0.00	0.00	− 0.20	0.01	0.00
Orthodox	− 0.18	0.02	0.00	− 0.18	0.02	0.00	− 0.12	0.02	0.00	− 0.10	0.02	0.00	− 0.09	0.02	0.00
Other Protestant	0.08	0.02	0.00	0.08	0.02	0.00	0.08	0.02	0.00	− 0.03	0.02	0.24	0.02	0.02	0.49
Other Christian	− 0.30	0.02	0.00	− 0.32	0.02	0.00	− 0.29	0.02	0.00	− 0.43	0.02	0.00	− 0.40	0.02	0.00
Catholic	− 0.32	0.06	0.00	− 0.31	0.06	0.00	− 0.08	0.07	0.24	− 0.09	0.07	0.16	− 0.07	0.07	0.31
Islam	0.66	0.11	0.00	0.68	0.11	0.00	1.10	0.13	0.00	0.79	0.12	0.00	0.86	0.12	0.00
Eastern	− 0.15	0.12	0.19	− 0.13	0.12	0.28	− 0.01	0.13	0.93	− 0.01	0.12	0.91	0.04	0.13	0.75
Jewish	− 0.22	0.15	0.14	− 0.21	0.15	0.15	0.11	0.17	0.51	− 0.02	0.16	0.90	0.00	0.16	0.99
*Men*															
State church	Ref			Ref			Ref			Ref			Ref		
No religion	− 0.22	0.00	0.00	− 0.22	0.00	0.00	− 0.17	0.00	0.00	− 0.14	0.00	0.00	− 0.11	0.01	0.00
Orthodox	− 0.15	0.02	0.00	− 0.15	0.02	0.00	− 0.09	0.02	0.00	− 0.07	0.02	0.00	− 0.06	0.02	0.00
Other Protestant	0.22	0.02	0.00	0.20	0.02	0.00	0.20	0.02	0.00	0.01	0.02	0.74	0.03	0.02	0.20
Other Christian	− 0.17	0.03	0.00	− 0.13	0.03	0.00	− 0.10	0.03	0.00	− 0.36	0.02	0.00	− 0.35	0.02	0.00
Catholic	− 0.18	0.05	0.00	− 0.22	0.05	0.00	− 0.05	0.06	0.41	− 0.10	0.06	0.09	− 0.08	0.06	0.14
Islam	0.00	0.14	0.99	− 0.03	0.14	0.83	0.19	0.16	0.21	0.13	0.15	0.37	0.15	0.15	0.32
Eastern	− 0.25	0.11	0.02	− 0.28	0.11	0.01	− 0.18	0.11	0.12	− 0.28	0.11	0.01	− 0.26	0.11	0.02
Jewish	0.05	0.15	0.74	− 0.02	0.15	0.91	0.24	0.17	0.16	0.24	0.17	0.16	0.24	0.17	0.14

Number of women is 629,038 and number of men is 650,044. The average marginal effects are based on estimates from Poisson regression models. Model 1 contains no control variables. Model 2 includes birth cohort, mother tongue, and educational level. Models 3, 4, and 5 stepwise add municipality of residence, marital status, and change of religious denomination (at age 45 vs. age 15)

**Fig. 3:**
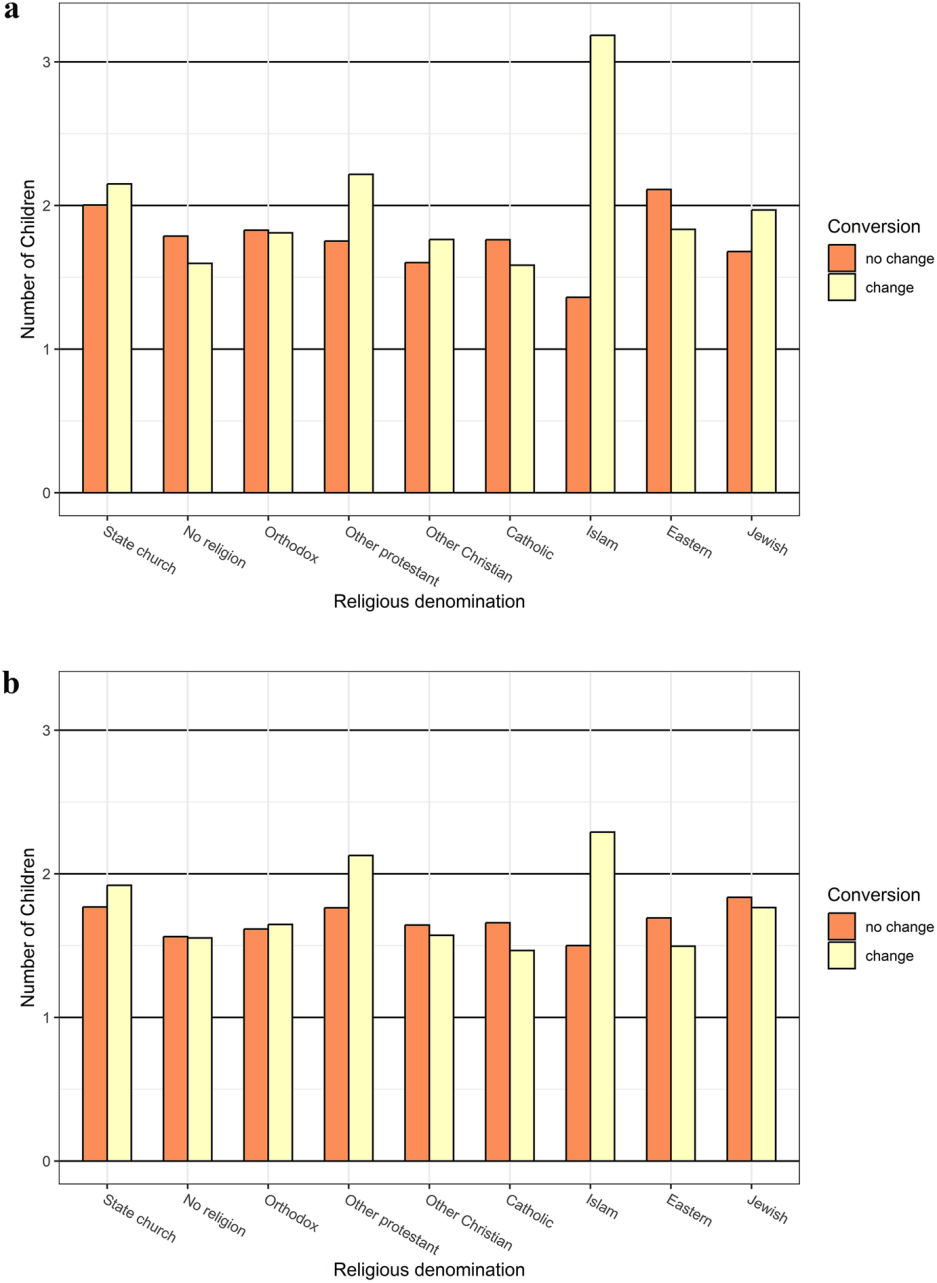
Mean number of children at age 45 by religious denomination, for those who had not and those who had changed religion since age 15, women (a, top panel) and men (b, bottom panel). The group who has changed religion includes everyone with at least two different denominations observed in our data. We categorize them by their last observed religion

For both women and men, converts to the state church, other Protestants, and Muslims have higher fertility than non-converts, while converts to Catholicism and Eastern denominations have lower fertility than non-converts (Fig. 3). The difference between converts and non-converts is particularly marked for Islam, albeit driven by few individuals. Women who became non-affiliated with any religion, which primarily means that they dissociated from the state church, have about 0.2 fewer children than those who remained affiliated with the state church. This secularization effect is smaller in men, or close to zero. Converts are more distinct from the average population than their non-convert counterparts, both when the group they convert to is larger, including no religious denomination, and when it is smaller. Table A1 in the Appendix shows that variations in fertility across converts and non-converts are, to a minor extent, related to birth cohort, mother tongue, and educational level. Controlling for marital status reduces fertility differences across the three largest affiliation, suggesting that some of the fertility effect is related to selection into marriage. In contrast, controlling for marital status has no such effect for smaller religions, and denominations with fertility with lower than average fertility has even lower fertility, while denominations with higher than average fertility have even higher fertility. Results in Fig. 3 show converts by their destination religion. In Table A2 in the Appendix, we show the religious origin (at age 15) and destination (at age 45) of religious converts. It shows considerable inflow and outflow of all the three largest denominations.

**Fig. 4:**
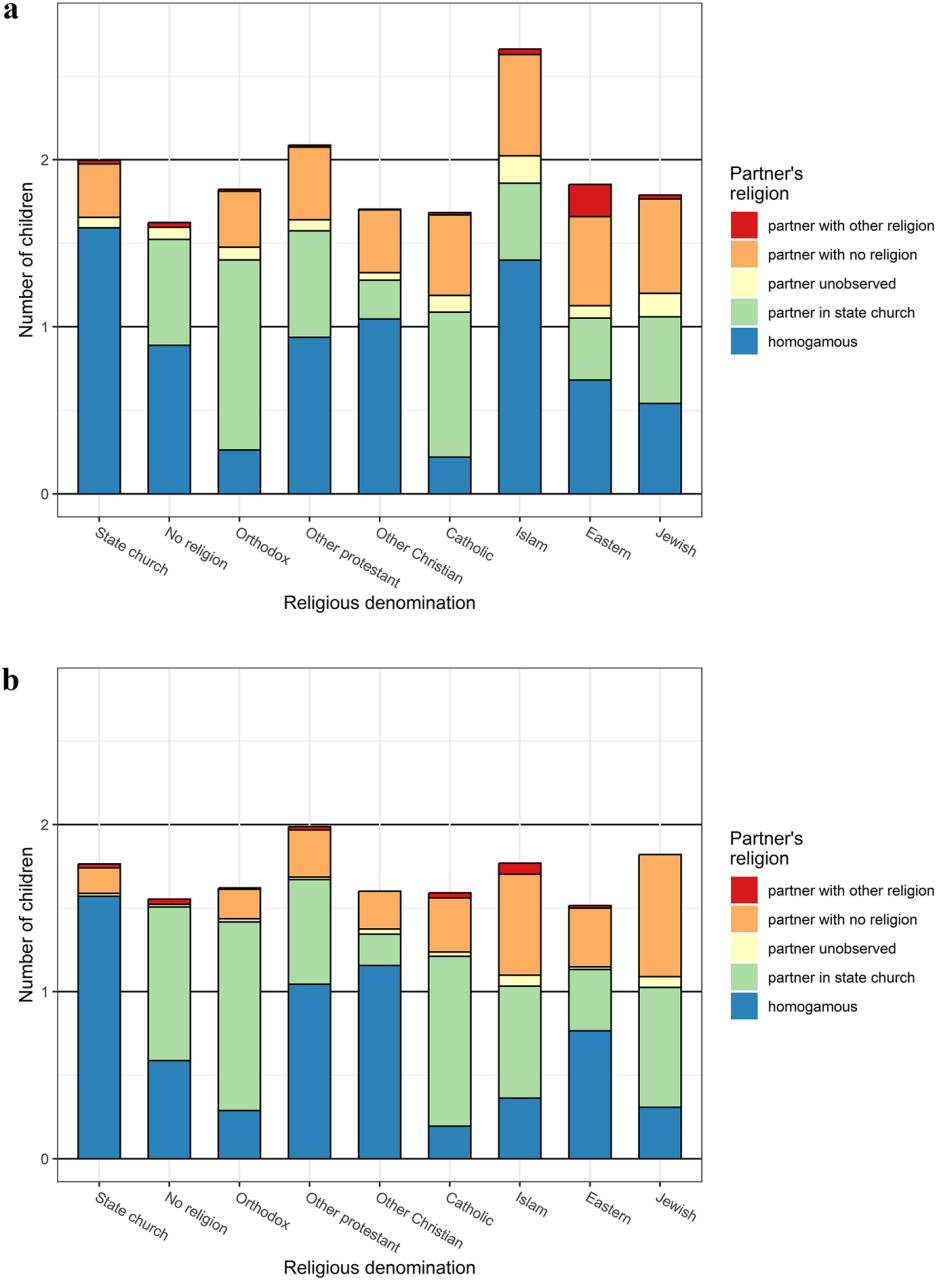
Mean number of children at age 45 by religious denomination, broken down by the first childbearing partner’s religious denomination, women (a, top panel) and men (b, bottom panel). We attribute each birth an index person to the partner for that birth and the partner’s religious affiliation, and categorize them into five groups based on their partner’s observed religion

Figure 4 breaks average fertility by religious denomination down by the first childbearing partner’s religious denomination. For almost all groups, a larger part of women’s than men’s fertility is with a partner with no religious denomination. A larger part of men’s fertility than women’s fertility, on the other hand, is with a female partner who belongs to the state church. These sex differentials largely reflect that more women than men belong to the state church (80.9% vs. 72.6% for the study population), while fewer women than men are religiously non-affiliated (16.9% vs. 25.6%). The highest share of fertility related to homogamous partnerships is found for women of the state church, followed by Islam and other Christians. For men, homogamous partnerships’ contribution to fertility is highest for members of the state church, followed by other Christians and other Protestants. For both women and men, the lowest contribution of religious homogamy to fertility is among Catholics, Orthodox, and Jews. The largest sex difference on this account is for the few members of Islam.

For both women and men, having a partner with a discordant religious denomination is associated with lower fertility, or about 0.15 fewer children. This is shown in Table 3 (Model 1), where the study population has been restricted to persons with at least one child and for whom the first childbearing partner can be identified in the registers. The association is largely unaffected by birth cohort, mother tongue, educational level (Model 2), municipality of residence (Model 3), and change of religious denomination (Model 5), while the inclusion of marital status (Model 4) reduces the mean difference to 0.10 fewer children, suggesting that some of the fertility suppressing effect is related to a higher probability of not being married. Having had more than one childbearing partner is associated with higher fertility, or roughly one more child, and this association is slightly smaller for women than for men. Fertility differences by religious denomination are generally larger for women than for men. Relatedly, fertility differences by partner’s religious denomination are generally larger for men than for women. For both female and male fertility, religious denomination is thus more important among women than men.

**Table 3:** Average marginal effects (with standard errors and p-values) of religious denomination of ego and the partner at age 45, whether they have the same religious denomination at age 45, and number of ego’s childbearing partners, on the number of children at age 45, by sex

	Model 1			Model 2			Model 3			Model 4			Model 5		
	dy/dx	S.E	P >|z|	dy/dx	S.E	P >|z|	dy/dx	S.E	P >|z|	dy/dx	S.E	P >|z|	dy/dx	S.E	P >|z|
*Women*															
Ego’s religious denomination															
State church	Ref			Ref			Ref			Ref			Ref		
No religion	− 0.15	0.01	0.00	− 0.16	0.01	0.00	− 0.12	0.01	0.00	− 0.10	0.01	0.00	− 0.04	0.01	0.00
Orthodox	− 0.01	0.02	0.70	− 0.01	0.02	0.78	0.02	0.02	0.50	0.00	0.02	0.99	0.02	0.02	0.39
Other Protestant	0.22	0.03	0.00	0.21	0.03	0.00	0.20	0.03	0.00	0.14	0.03	0.00	0.20	0.04	0.00
Other Christian	0.17	0.05	0.00	0.16	0.05	0.00	0.16	0.05	0.00	0.07	0.05	0.16	0.12	0.05	0.02
Catholic	− 0.01	0.09	0.93	0.00	0.09	1.00	0.12	0.09	0.19	0.09	0.09	0.33	0.12	0.09	0.19
Islam	0.78	0.15	0.00	0.78	0.15	0.00	0.91	0.16	0.00	0.81	0.15	0.00	0.88	0.15	0.00
Eastern	0.24	0.19	0.21	0.24	0.19	0.21	0.30	0.19	0.12	0.26	0.19	0.16	0.34	0.19	0.08
Jewish	0.09	0.21	0.68	0.09	0.21	0.69	0.22	0.23	0.33	0.13	0.22	0.56	0.15	0.22	0.49
Partner’s religious denomination															
State church	Ref			Ref			Ref			Ref			Ref		
No religion	− 0.07	0.01	0.00	− 0.07	0.01	0.00	− 0.03	0.01	0.00	− 0.03	0.01	0.00	− 0.03	0.01	0.00
Orthodox	− 0.03	0.02	0.22	− 0.03	0.03	0.30	− 0.01	0.03	0.79	− 0.02	0.03	0.46	− 0.01	0.03	0.59
Other Protestant	0.21	0.04	0.00	0.22	0.04	0.00	0.22	0.04	0.00	0.18	0.04	0.00	0.18	0.04	0.00
Other Christian	0.09	0.05	0.08	0.09	0.05	0.10	0.12	0.05	0.03	0.08	0.05	0.12	0.08	0.05	0.13
Catholic	− 0.10	0.06	0.08	− 0.10	0.06	0.09	0.02	0.06	0.77	0.00	0.06	0.94	0.00	0.06	0.98
Islam	− 0.11	0.09	0.23	− 0.11	0.09	0.22	0.01	0.10	0.92	− 0.02	0.09	0.86	− 0.01	0.09	0.90
Eastern	− 0.19	0.18	0.31	− 0.18	0.18	0.33	− 0.16	0.19	0.40	− 0.19	0.18	0.30	− 0.19	0.18	0.30
Jewish	0.01	0.17	0.94	0.02	0.17	0.92	0.15	0.18	0.38	0.14	0.17	0.43	0.15	0.17	0.39
Same religious denomination															
Yes	Ref			Ref			Ref			Ref			Ref		
No	− 0.16	0.01	0.00	− 0.16	0.01	0.00	− 0.14	0.01	0.00	− 0.10	0.01	0.00	− 0.10	0.01	0.00
Number of childbearing partners															
1	Ref			Ref			Ref			Ref			Ref		
2+	0.93	0.01	0.00	0.91	0.01	0.00	0.93	0.01	0.00	0.97	0.01	0.00	0.97	0.01	0.00
*Men*															
Ego’s religious denomination															
State church	Ref			Ref			Ref			Ref			Ref		
No religion	− 0.08	0.01	0.00	− 0.09	0.01	0.00	− 0.05	0.01	0.00	− 0.05	0.01	0.00	0.01	0.01	0.40
Orthodox	− 0.01	0.03	0.61	− 0.02	0.03	0.57	0.01	0.03	0.81	− 0.01	0.03	0.74	0.00	0.03	0.95
Other Protestant	0.23	0.04	0.00	0.23	0.04	0.00	0.23	0.04	0.00	0.15	0.04	0.00	0.19	0.04	0.00
Other Christian	0.11	0.06	0.04	0.12	0.06	0.03	0.16	0.06	0.01	0.11	0.06	0.05	0.15	0.06	0.01
Catholic	− 0.02	0.08	0.82	− 0.04	0.08	0.66	0.08	0.08	0.33	0.03	0.08	0.73	0.05	0.08	0.55
Islam	− 0.03	0.19	0.88	− 0.04	0.19	0.81	0.09	0.20	0.65	0.07	0.20	0.74	0.10	0.20	0.63
Eastern	0.00	0.18	0.99	− 0.02	0.18	0.91	0.01	0.18	0.97	− 0.05	0.17	0.76	0.01	0.18	0.97
Jewish	0.15	0.22	0.49	0.13	0.22	0.54	0.29	0.23	0.20	0.25	0.23	0.27	0.26	0.23	0.24
Partner’s religious denomination															
State church	Ref			Ref			Ref			Ref			Ref		
No religion	− 0.17	0.01	0.00	− 0.17	0.01	0.00	− 0.14	0.01	0.00	− 0.12	0.01	0.00	− 0.12	0.01	0.00
Orthodox	− 0.10	0.02	0.00	− 0.10	0.02	0.00	− 0.08	0.02	0.00	− 0.09	0.02	0.00	− 0.09	0.02	0.00
Other Protestant	0.17	0.03	0.00	0.17	0.03	0.00	0.16	0.03	0.00	0.12	0.03	0.00	0.12	0.03	0.00
Other Christian	0.11	0.05	0.04	0.12	0.05	0.03	0.11	0.05	0.04	0.02	0.05	0.69	0.02	0.05	0.69
Catholic	− 0.22	0.05	0.00	− 0.24	0.05	0.00	− 0.15	0.06	0.01	− 0.20	0.06	0.00	− 0.20	0.06	0.00
Islam	0.10	0.17	0.54	0.11	0.17	0.52	0.20	0.17	0.26	0.21	0.18	0.22	0.21	0.18	0.22
Eastern	− 0.23	0.12	0.06	− 0.22	0.12	0.06	− 0.19	0.12	0.13	− 0.24	0.12	0.04	− 0.24	0.12	0.05
Jewish	− 0.03	0.19	0.88	− 0.06	0.19	0.76	0.07	0.20	0.71	0.03	0.19	0.88	0.03	0.19	0.86
Same religious denomination															
Yes	Ref			Ref			Ref			Ref			Ref		
No	− 0.15	0.01	0.00	− 0.15	0.01	0.00	− 0.14	0.01	0.00	− 0.10	0.01	0.00	− 0.10	0.01	0.00
Number of childbearing partners															
1	Ref			Ref			Ref			Ref			Ref		
2+	1.08	0.01	0.00	1.10	0.01	0.00	1.14	0.01	0.00	1.17	0.01	0.00	1.17	0.01	0.00

Number of women is 494,802 and number of men is 472,789. The average marginal effects are based on estimates from Poisson regression models. Model 1 contains no control variables. Model 2 includes birth cohort, mother tongue, and educational level. Models 3, 4, and 5 stepwise add municipality of residence, marital status, and change of religious denomination (at age 45 vs. age 15). All results refer to egos with at least one child and for whom the first childbearing partner is identified in the registers

In most cases, except for denominations with few members, one’s partner’s religious denomination has the same association with fertility as one’s own religious denomination. Effect sizes for religious denominations are, in general, notably smaller among these partnered individuals than in the overall population (cf. Table 2), which largely has to do with the substantial differences in childlessness by religious denomination. When control variables are included, most of the estimates for religious denominations of individuals and their partners are either close to zero or statistically not significant. However, there are some exceptions. As compared with female members of the state church, other Protestant women have 0.20 more children, other Christian women 0.12 more, and Islamic women 0.88 more (Model 5). For women, having a partner in the “other Protestant” group is associated with 0.18 more children. Compared with male members of the state church, other Protestant men have 0.19 more children, and other Christian men have 0.15 more children. For men, having a partner who is non-affiliated is associated with 0.12 fewer children, Orthodox with 0.09 fewer children, other Protestant with 0.12 more children, Catholic with 0.20 fewer children, and Eastern with 0.24 fewer children.

## Discussion

We have identified heterogeneity by religion in completed fertility in Finland. Overall, we found a clear divide between secular and non-secular individuals, which is consistent with most previous research on religion and fertility (Berman et al., 2018; Frejka & Westoff, 2008; Hackett, 2008; Peri-Rotem, 2016). For understanding Finnish fertility, lower fertility among the non-affiliated is the most consequential pattern we observed. In contrast, most other religious groups constitute a comparatively small share of the population in Finland. We found that most other religions have somewhat lower fertility than members of the Finnish state church. This is in contrast to much international research (Berman et al., 2018; Hackett, 2008) that has associated religions such as Islam, Evangelical Protestantism, Catholicism, and Mormonism with higher fertility than mainline Protestantism, whose members can be considered comparable with (at least the mostly secular) members of the Finnish state church. Overall, however, differences between denominations are rather modest, and the similarity is perhaps more striking than the differences.

Our findings are a novel contribution in that we provide longitudinal life course data on both religion and fertility. This allowed us to examine how changing religion over one’s life course affects fertility. The differences by religion are in most cases rather small, but we found that converts of most religions are typically somewhat more distinct from the general population than non-converts. One explanation for such patterns may be that while for large groups in society their religious beliefs are a quite routine part of their lives, shared with their friends and family, for converts religion may be more salient as it represents a break with their upbringing. As conversion is based on an individual choice, any pro-natalist or anti-natalist features of the religion they convert to may thus be expressed more clearly.

Our research design allowed us to break down individuals’ fertility histories by both their own parity and their partner’s religions. We could thus document how homogamy and heterogamy differ across religious groups and affect fertility by religious denomination. The prevalence of homogamy is predictably affected by the sizes of the different religious groups, as small groups are less likely to have a homogamous partner, but this explains far from all the patterns in how partners’ religions and religious homogamy contribute to fertility differences by religious denomination. Higher childlessness among smaller religious groups may also be affected by difficulties in finding a homogamous partner.

We have studied religion as measured by official membership in a government-recognized religious organization. This is both a limitation and a strength of our design. An obvious limitation is that it says little about how individual religiosity, as distinct from religious affiliation, affects fertility, which has been a focus of much research on fertility and religion. The primary clear-cut inference related to the intensity of religiosity is that the non-affiliated population represents much fewer religious individuals than members of the state church, which is also likely why we generally observe large fertility differentials between these two groups (see analyses with survey data in supplemental text S1). The members of other Protestant affiliations not linked to the state church consist of individuals with, on average, higher religiosity. A major strength of our approach is that the data used have no traditional measurement errors, sampling errors, or other missing information. They are also longitudinal, meaning that we measured both fertility and religious denomination in every subsequent year for the complete population for over 50 years.

 Our data is of very high quality for older established denominations, while it is less comprehensive for newer religious organizations. This mean that for some individuals, their religious lives likely occurred outside the institutions recognized in our data. This is likely more common for recent immigrants to Finland (who were excluded from the study), while rather uncommon for the much larger native Finnish-born population (who were included). For most individuals in the three largest denominations here—members of the state church, the non-affiliated, and the Orthodox—the religious denomination is the outcome of a deliberate choice and has clear sociological meaningfulness. Being a member of the state church is associated with substantial additional income tax payments over devotees’ lifetimes, and most individuals are unlikely to remain members unless they share at least some affiliation with the state church, though this connection is likely often based on notions of cultural affinity rather than faith-based reasons (Iversen, 2006; Xia et al., 2023). We thus measured something sociologically meaningful, and our supplemental survey analyses indeed show very large differences in self-rated religiosity across state church members and non-affiliated, even though intense religious practice such as weekly church attendance is uncommon. A possible reason for the higher fertility among state church members is that the Finnish state church may serve as a symbol for community cohesion, stability, and traditional and national values in a Finnish context (Church Research Institute, 2005). Community cohesion has been described as important to understanding trends in secularization in Finland (Xia et al., 2023). Affinity toward such preferences and values, and consequently state church membership, may be associated with family life and fertility. This could explain why most other non-majority choices then typically display lower fertility than members of the state church.

As a mirror image, the non-affiliated represent a minority in Finland, comprising individuals who have actively chosen not to be members of the state church, and that choice is likely associated with a set of values linked to strong ideals of secularization. To truly understand the situation for members of smaller religious minorities in Finland, and before deriving major implications for other nations where similar religions contribute to a much larger share of the national population, a careful consideration of the cultural and historical context and immigration history of each group is appropriate. However, this lies beyond the scope of the present study (which had focused on completed fertility using observed life course data), as foreign-born immigration to Finland has mostly occurred since year 2000. A different interesting aspect of fertility and union formation is related to when in the life course events occur, which is an interesting future area to explore.

Our paper serves as a novel contribution to the overall study of religion and fertility through its novel application of administrative registers. In some way, Finland is unlike many other countries in the world, being largely secular but religiously comparatively homogenous, particularly when disregarding the recent inflow of foreign-born immigrants, who cannot be fully observed with regard to completed childbearing. Regardless, many of the patterns we observed have, to our knowledge, not been documented previously, and they can inform research on religion and fertility in other contexts, where religious affiliation may be a powerful determinant of population-level fertility. We also think that many of our findings challenge societal beliefs and stereotypes on the link between fertility and religion, and thus should be of interest to a wide variety of researchers and policy makers.

## Supplementary Information

Below is the link to the electronic supplementary material.

Supplementary text S1 (PDF 656 kb)

Supplementary tables S2 (PDF 376 kb)

Supplementary file S3 (XLSX 22 kb)

## Data Availability

The data are available inside the FIONA remote access system, and only aggregated data are allowed to be exported from the system. Other researchers can access the underlying micro-level data if they apply for necessary ethical approvals from Statistics Finland, but it can otherwise not be shared as it contains sensitive personal information for the complete population of Finland. All the aggregated output used to make the figures in the paper are available in a supplemental file S3.
