# Supplementary material for: Religion and Fertility: A Longitudinal Register Study Examining Differences by Sex, Parity, Partner’s Religion, and Religious Conversion in Finland
Source: Eur J Popul. 2024 Feb 19;40(1):9. doi: 10.1007/s10680-023-09693-0 (PMC10876502; doi:10.1007/s10680-023-09693-0)

Supplemental text 1 – A survey of religious beliefs and practices in the Finnish population, using data from the European Social Survey for 6 surveys from the years 2010 to 2020.

A supplemental text for the manuscript “Religion and fertility – A longitudinal register study examining differences by sex, parity, partner’s religion, and religious conversion in Finland”.

To assess the extent of religiosity in Finland we used 6 waves of survey data from the European Social Survey (ESS EIRC, 2023). It was collected every 2 years between 2010 and 2020 using independent national representative samples of individuals 15 and above. Pooling 6 waves we got a sample size of 11419 individuals allowing us to get meaningful responses also for less common religions. In waves 6 to 10 of the ESS people could self-identify with a religion, using a set of Finland-specific religious categories (other waves used a European-wide classification). The categories map well to how we have organized the formal religious organizations in our administrative data.

The response to the ESS overlaps relatively well with the distribution of our administrative data (See Table S-T 1 below). The ESS population and the cohorts in the administrative registers are quite different in both age, cohorts, and most importantly length of residence in Finland, from the data in our manuscript on religion and fertility in Finland. The ESS data being nationally representative in the 2010s include many more immigrants that are not part of the target population of our national data. The ESS data, being a survey, may also suffer from non-representative responses in some groups.

The major difference is that a substantial share of members of the National Evangelical Lutheran Church choose to differ as not belonging to a religion when asked in social science survey. This amounts to perhaps a quarter of members of the national church who choose to self-identify as not belonging to any religion. Other minor discrepancies are smaller shares of orthodox, and higher shares of minor religious denominations, though this may be due to the different population definitions.

We examined the intensity of religious self-identification (“How religious are you”, on a scale from 0 to 10, with 10 being the highest), personal religious practices (“How often do you pray”, on a 7-category scale), and organized religious activities (“How often do you attend religious practices, except at special occasions, on a 7-category scale), using 3 different questions from the ESS data. The results are presented in 3 figures below (Figure S-T 1, S-T 2, & S-T 3).

To summarize the findings, religious self-identification is very high across all religious denominations, except the nones (figure S-F 1). Among the members of the national church – that are often described as secular – 47% describe themselves as very religious (responding 7-10), and another 36% describe themselves as quite religious (responding 4-6). In contrast, only 2.5% chose the most religious category among the nones, and 31% chose the least religious category (0-1), and another 21% chose the category 2-3. In the ESS data, the differences between state church members and nones are thus very salient. Religiosity among other denominations is higher than the state church, but not dramatically so.

When we examine how often people pray (which importantly is different in importance across religious traditions), we find larger differences (figure S-F 2). Still, the difference between the state church and the nones is very marked. We do however find a much higher prevalence of frequent praying among the smaller Christian denominations, supportive of higher shares of practicing religious members in these denominations. Quite substantive shares of individuals in the state church do however report frequent praying, which means it is not uncommon in the Finnish population.

Finally, we examine attendance of religious practice, and here we find very large differences across the different denominations (figure S-F 3). While those identifying with the state church often report some rare church attendance, frequent church attendance is very uncommon, in line with descriptions of many state church members as not practicing Christians. In contrast, church attendance is very high among the smaller Christian denominations. The Orthodox, Catholics, and Muslims report intermediate levels of church attendance, where some attend regularly and many do not.

The results are consistent with survey responses from 2003 on beliefs in key Christian tenants in the Finnish population (Church Research Institute, 2005), where 36% reported firmly believing that "Jesus rose from the dead", 25% deemed it likely, 18% deemed it unlikely, and 16% did not believe this at all. Similar distributions of answers were given to the questions "God created the world", "Jesus was born of a virgin", and "Jesus is the Son of God".

In a related survey from 2003, 73% reported considering themselves Christians, 63% reported considering themselves Lutherans, 38% reported considering themselves religious, and 12% reported considering themselves atheists (Church Research Institute, 2005).

Taken together the results from our ESS results suggest that considering yourself religious and Christian is common in the Finnish population. These beliefs are much more common among state church members than non-affiliated. Own religious practice such as praying is also relatively common. Thus, the results are consistent with that being a member of the state church is a highly salient individual characteristic, and that their spiritual beliefs and practices clearly differ from those that do not self-identify with a religion in the ESS data. It is therefore perhaps not surprising that we find differences in demographic behavior. On the other hand, intense everyday religious practices are low among most members of Finnish society, including members of the state church. Consequently, the characterization of Finland as a secular society appears to be broadly correct. The results are also consistent with that religious intense practice is much more common, and intense among smaller Protestants and other Christian denominations in Finland, as often suggested in overviews of the Nordic religious landscape (Iversen, 2006).

The findings of high proportions reporting high religiosity in the state church may also partially explain why a large share of the Finnish population is content with paying church taxes, even though their active daily religious activities are quite uncommon, as they self-identify with the Church.

All the interpretations here should be considered with some care, as the population and definitions based on survey responses differ from the population we identify with national administrative data. In particular, people formally belonging to the state church, and paying church taxes, will include a majority of those identified as national Lutherans in the ESS, but it will also include substantial shares of people that identify as non-religious in the ESS. The ESS data will also include larger shares of both older and younger cohorts compared to those born between 1956 to 1975 in our manuscript with administrative data.

References:

Church Research Institute. (2005). Church in change (Vol. 2000/03). Tampere: Research Institution of the Evangelical Lutheran Church of Finland.

European Social Survey European Research Infrastructure (ESS ERIC). (2023). ESS10 integrated file, edition 3.1 [Data set]. Sikt - Norwegian Agency for Shared Services in Education and Research. https://doi.org/10.21338/ess10e03_1

Iversen, H. R. (2006). Religious Profile of the Nordic. In H. R. Iversen (Ed.), Rites of Ordination and Commitment in the Churches of the Nordic Countries: Theology and Terminology (pp. 35-39): University of Chicago Press.

Table S-T 1: Response from the European Social Survey to the question "Which religion or denomination do you belong at present"? Pooled over Wave 5-10, in Finland 2010-2020. Number of respondents by denomination.

	Evangelical Lutheran	Eastern Orthodox	Roman Catholic	Protestant Free Churches	Other christians	Islam	Other non-christians	Nones	Other responses
N	5583	114	22	151	61	60	45	5279	104
%	48.9	1.0	0.2	1.3	0.5	0.5	0.4	46.2	0.9

Figure S-T 1

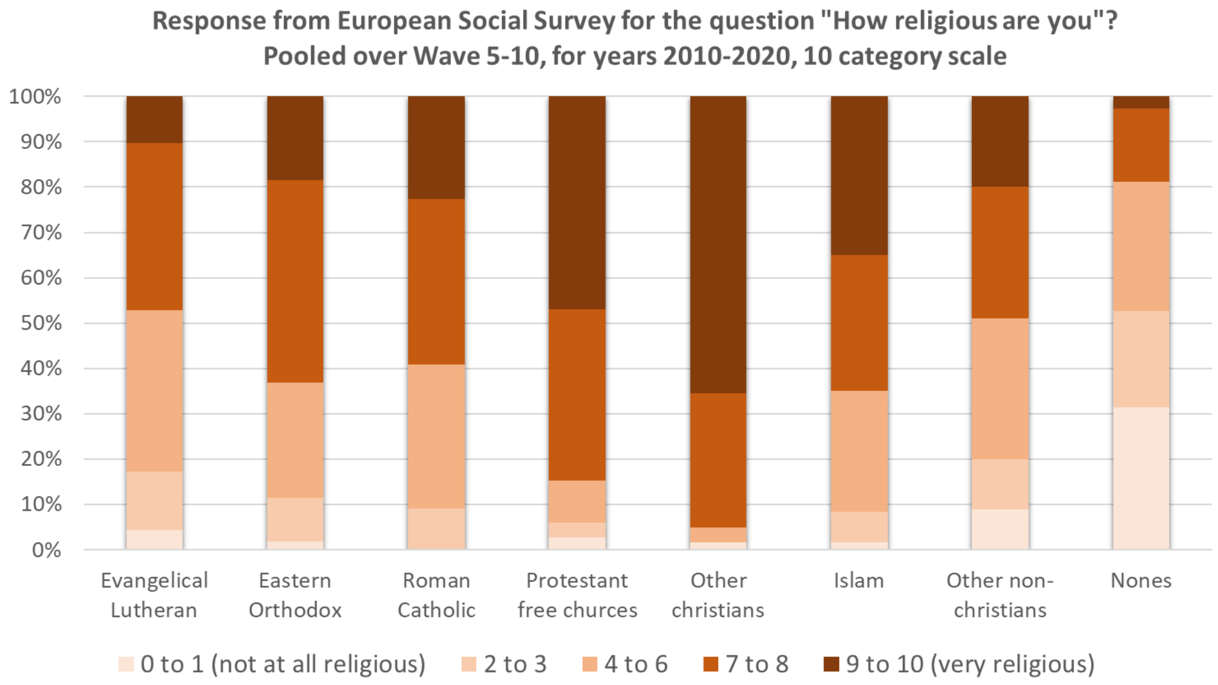

Figure S-T 2

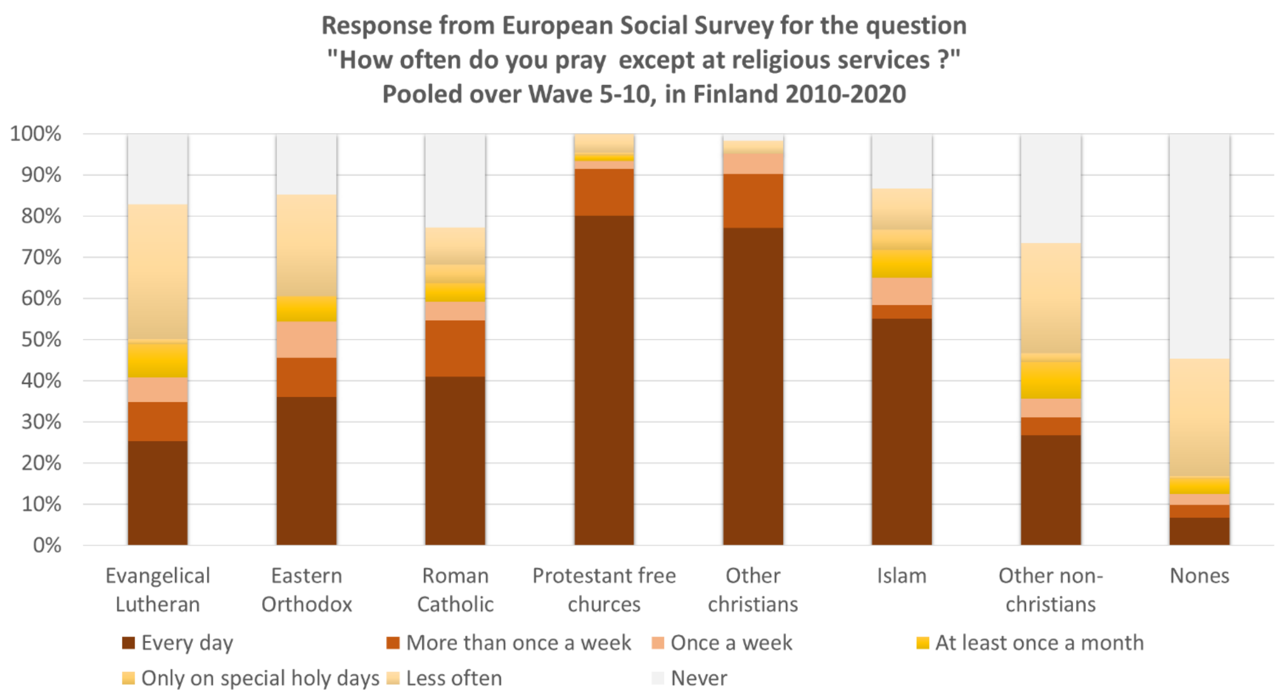

Figure S-T 3

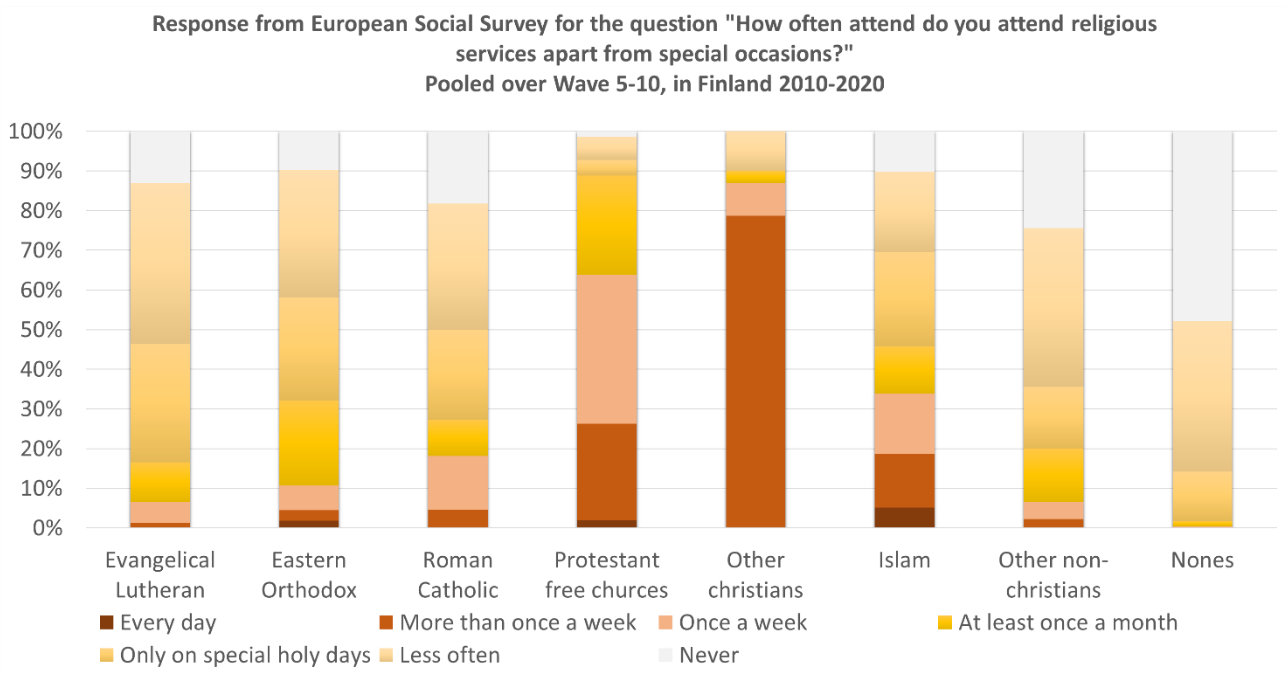

Supplement: Supplementary file 1 — Supplementary text S1 (PDF 656 kb) [file 10680_2023_9693_MOESM1_ESM.pdf]
