# Supplementary material for: Religion and Fertility: A Longitudinal Register Study Examining Differences by Sex, Parity, Partner’s Religion, and Religious Conversion in Finland
Source: Eur J Popul. 2024 Feb 19;40(1):9. doi: 10.1007/s10680-023-09693-0 (PMC10876502; doi:10.1007/s10680-023-09693-0)
Supplement: Supplementary file 2 — Supplementary tables S2 (PDF 376 kb) [file 10680_2023_9693_MOESM2_ESM.pdf]

APPENDIX

Table A1. Average marginal effects (with standard errors and p-values) of change of religious denomination on the number of children at age 45, by sex, cohorts born 1956-1975

	Model 1			Model 2			Model 3			Model 4		
	dy/dx	S.E.	P>|z|	dy/dx	S.E.	P>|z|	dy/dx	S.E.	P>|z|	dy/dx	S.E.	P>|z|
WOMEN												
State church, no change	Ref.			Ref.			Ref.			Ref.		
State church, change	0.15	0.02	0.00	0.13	0.02	0.00	0.20	0.02	0.00	0.10	0.02	0.00
No religion, no change	-0.22	0.01	0.00	-0.22	0.01	0.00	-0.11	0.01	0.00	-0.09	0.01	0.00
No religion, change	-0.41	0.00	0.00	-0.40	0.00	0.00	-0.33	0.00	0.00	-0.28	0.00	0.00
Orthodox, no change	-0.17	0.02	0.00	-0.18	0.02	0.00	-0.13	0.02	0.00	-0.08	0.02	0.00
Orthodox, change	-0.19	0.03	0.00	-0.18	0.03	0.00	-0.08	0.03	0.02	-0.14	0.03	0.00
Other protestant, no change	-0.25	0.04	0.00	-0.25	0.04	0.00	-0.23	0.04	0.00	-0.25	0.04	0.00
Other protestant, change	0.21	0.03	0.00	0.22	0.03	0.00	0.20	0.03	0.00	0.06	0.03	0.03
Other Christian, no change	-0.40	0.04	0.00	-0.42	0.04	0.00	-0.37	0.04	0.00	-0.51	0.04	0.00
Other Christian, change	-0.24	0.03	0.00	-0.26	0.03	0.00	-0.24	0.03	0.00	-0.38	0.03	0.00
Catholic, no change	-0.24	0.08	0.00	-0.24	0.08	0.00	0.00	0.09	0.98	-0.02	0.09	0.86
Catholic, change	-0.42	0.09	0.00	-0.40	0.09	0.00	-0.18	0.10	0.07	-0.19	0.10	0.05
Islam, no change	-0.64	0.15	0.00	-0.59	0.16	0.00	-0.34	0.18	0.06	-0.21	0.20	0.28
Islam, change	1.18	0.14	0.00	1.17	0.14	0.00	1.64	0.17	0.00	1.09	0.14	0.00
Eastern, no change	0.11	0.48	0.82	0.14	0.49	0.78	0.21	0.50	0.68	0.17	0.49	0.72
Eastern, change	-0.17	0.12	0.16	-0.15	0.12	0.23	-0.03	0.13	0.84	-0.03	0.13	0.83
Jewish, no change	-0.32	0.18	0.07	-0.32	0.18	0.07	-0.01	0.21	0.96	-0.11	0.20	0.57
Jewish, change	-0.03	0.25	0.89	-0.02	0.25	0.94	0.33	0.29	0.26	0.13	0.27	0.62
MEN												
State church, no change	Ref.			Ref.			Ref.			Ref.		
State church, change	0.15	0.02	0.00	0.17	0.02	0.00	0.24	0.02	0.00	0.06	0.02	0.00
No religion, no change	-0.21	0.01	0.00	-0.19	0.01	0.00	-0.11	0.01	0.00	-0.08	0.01	0.00
No religion, change	-0.22	0.00	0.00	-0.22	0.00	0.00	-0.17	0.00	0.00	-0.14	0.00	0.00
Orthodox, no change	-0.15	0.02	0.00	-0.15	0.02	0.00	-0.09	0.02	0.00	-0.06	0.02	0.01
Orthodox, change	-0.12	0.04	0.01	-0.15	0.04	0.00	-0.06	0.04	0.21	-0.13	0.04	0.00
Other protestant, no change	-0.01	0.04	0.87	-0.02	0.04	0.54	0.00	0.04	0.92	-0.06	0.04	0.10
Other protestant, change	0.36	0.03	0.00	0.34	0.03	0.00	0.33	0.03	0.00	0.05	0.03	0.10
Other Christian, no change	-0.13	0.04	0.00	-0.08	0.04	0.05	-0.04	0.04	0.35	-0.25	0.04	0.00
Other Christian, change	-0.20	0.03	0.00	-0.17	0.03	0.00	-0.15	0.03	0.00	-0.44	0.03	0.00
Catholic, no change	-0.11	0.07	0.11	-0.15	0.07	0.03	0.04	0.07	0.61	-0.04	0.07	0.57
Catholic, change	-0.30	0.09	0.00	-0.34	0.09	0.00	-0.19	0.09	0.04	-0.20	0.09	0.03
Islam, no change	-0.27	0.16	0.09	-0.32	0.15	0.04	-0.12	0.17	0.49	-0.09	0.18	0.61
Islam, change	0.52	0.27	0.06	0.57	0.28	0.04	0.86	0.31	0.01	0.54	0.27	0.05
Eastern, no change	-0.08	0.36	0.83	-0.05	0.37	0.88	0.04	0.38	0.92	-0.15	0.34	0.66
Eastern, change	-0.27	0.11	0.02	-0.30	0.11	0.01	-0.20	0.12	0.10	-0.30	0.11	0.01
Jewish, no change	0.07	0.17	0.70	-0.01	0.17	0.96	0.26	0.19	0.17	0.32	0.20	0.10
Jewish, change	0.00	0.32	0.99	-0.03	0.32	0.92	0.18	0.35	0.61	-0.03	0.31	0.91

Number of women is 629,038 and number of men is 650,044.
The average marginal effects are based on estimates from Poisson regression models.
Model 1 contains no control variables. Model 2 includes Birth cohort, Mother tongue, and Educational level.
Models 3 and 4 stepwise add Municipality of residence and Marital status.
Change of religious denomination refers to religion at age 45 vs. religion at age 15.
Educational level, Mother tounge, Muncipality of residence, and Marital status refers to the situation at age 45.

Table A2-A. Religious denomination at age 45 versus at age 15, women and men, cohorts born 1956-1975

	WOMEN at age 45								
WOMEN at age 15	(1)	(2)	(3)	(4)	(5)	(6)	(7)	(8)	(9)
(1) State church	501,587	89,052	1,601	1,984	980	157	136	109	21
(2) No religion	6,493	16,048	152	614	899	61	14	16	11
(3) Orthodox	341	519	4,285	16	<10	<10	<10	<10	<10
(4) Other protestant	556	379	<10	1,014	<10	<10	<10	<10	<10
(5) Other Christian	111	333	<10	<10	1,043	<10	<10	<10	<10
(6) Catholic	25	25	<10	<10	<10	277	<10	<10	<10
(7) Islam	<10	<10	<10	<10	<10	<10	61	<10	<10
(8) Eastern	<10	<10	<10	<10	<10	<10	<10	<10	<10
(9) Jewish	<10	11	<10	<10	<10	<10	<10	<10	53

	MEN at age 45								
MEN at age 15	(1)	(2)	(3)	(4)	(5)	(6)	(7)	(8)	(9)
(1) State church	466,266	143,819	795	1,409	614	133	21	98	11
(2) No religion	4,996	21,128	70	585	729	56	<10	15	<10
(3) Orthodox	147	876	4,228	<10	<10	<10	<10	<10	<10
(4) Other protestant	337	406	<10	1,243	<10	<10	<10	<10	<10
(5) Other Christian	81	317	<10	<10	1,048	<10	<10	<10	<10
(6) Catholic	15	30	<10	<10	<10	352	<10	<10	<10
(7) Islam	<10	<10	<10	<10	<10	<10	60	<10	<10
(8) Eastern	<10	<10	<10	<10	<10	<10	<10	13	<10
(9) Jewish	<10	25	<10	<10	<10	<10	<10	<10	61

According to regulations by Statistics Finland, frequencies below ten are indicated with ‘<10’.

Table A3. Average marginal effects (with standard errors and p-values) of the control variables referred to in Table 2

	Model 2			Model 3			Model 4			Model 5		
	dy/dx	S.E.	P>|z|	dy/dx	S.E.	P>|z|	dy/dx	S.E.	P>|z|	dy/dx	S.E.	P>|z|
WOMEN												
Birth cohort (1956)	Ref.			Ref.			Ref.			Ref.		
1957	0.03	0.01	0.01	0.03	0.01	0.01	0.03	0.01	0.00	0.03	0.01	0.00
1958	0.04	0.01	0.00	0.04	0.01	0.00	0.06	0.01	0.00	0.06	0.01	0.00
1959	0.04	0.01	0.00	0.04	0.01	0.00	0.07	0.01	0.00	0.07	0.01	0.00
1960	0.05	0.01	0.00	0.06	0.01	0.00	0.10	0.01	0.00	0.10	0.01	0.00
1961	0.05	0.01	0.00	0.05	0.01	0.00	0.11	0.01	0.00	0.11	0.01	0.00
1962	0.04	0.01	0.00	0.05	0.01	0.00	0.12	0.01	0.00	0.12	0.01	0.00
1963	0.04	0.01	0.00	0.06	0.01	0.00	0.14	0.01	0.00	0.14	0.01	0.00
1964	0.03	0.01	0.01	0.05	0.01	0.00	0.14	0.01	0.00	0.14	0.01	0.00
1965	0.03	0.01	0.00	0.05	0.01	0.00	0.16	0.01	0.00	0.16	0.01	0.00
1966	0.02	0.01	0.07	0.04	0.01	0.00	0.15	0.01	0.00	0.15	0.01	0.00
1967	0.01	0.01	0.36	0.04	0.01	0.00	0.15	0.01	0.00	0.15	0.01	0.00
1968	0.03	0.01	0.00	0.06	0.01	0.00	0.17	0.01	0.00	0.17	0.01	0.00
1969	0.03	0.01	0.00	0.05	0.01	0.00	0.18	0.01	0.00	0.18	0.01	0.00
1970	0.01	0.01	0.30	0.03	0.01	0.01	0.16	0.01	0.00	0.16	0.01	0.00
1971	0.02	0.01	0.03	0.05	0.01	0.00	0.19	0.01	0.00	0.19	0.01	0.00
1972	0.05	0.01	0.00	0.06	0.01	0.00	0.20	0.01	0.00	0.20	0.01	0.00
1973	0.04	0.01	0.00	0.06	0.01	0.00	0.19	0.01	0.00	0.19	0.01	0.00
1974	0.03	0.01	0.00	0.06	0.01	0.00	0.20	0.01	0.00	0.20	0.01	0.00
1975	0.05	0.01	0.00	0.07	0.01	0.00	0.22	0.01	0.00	0.22	0.01	0.00
Mother tongue (Finnish)	Ref.			Ref.			Ref.			Ref.		
Swedish	0.07	0.01	0.00	0.05	0.01	0.00	0.08	0.01	0.00	0.08	0.01	0.00
Other	-0.14	0.06	0.02	-0.05	0.07	0.44	-0.02	0.07	0.80	-0.03	0.07	0.69
Educational level (Primary)	Ref.			Ref.			Ref.			Ref.		
Secondary	0.14	0.01	0.00	0.08	0.01	0.00	-0.01	0.01	0.12	-0.01	0.01	0.13
Tertiary, short-cycle	-0.02	0.01	0.02	-0.04	0.01	0.00	-0.19	0.01	0.00	-0.19	0.01	0.00
Tertiary, bachelor	0.05	0.01	0.00	0.04	0.01	0.00	-0.14	0.01	0.00	-0.14	0.01	0.00
Tertiary, master or higher	-0.05	0.01	0.00	0.00	0.01	0.59	-0.22	0.01	0.00	-0.22	0.01	0.00
Municipality of residence	No			Yes			Yes			Yes		
Marital status (Married)							Ref.			Ref.		
Not married							-1.32	0.00	0.00	-1.32	0.00	0.00
Divorced							-0.13	0.01	0.00	-0.13	0.01	0.00
Widow(er)							-0.17	0.02	0.00	-0.17	0.02	0.00
Change of religious denomination (No)										Ref.		
Yes										-0.06	0.01	0.00
MEN												
Birth cohort (1956)	Ref.			Ref.			Ref.			Ref.		
1957	0.02	0.01	0.10	0.02	0.01	0.06	0.03	0.01	0.00	0.03	0.01	0.00
1958	-0.01	0.01	0.61	0.00	0.01	0.71	0.02	0.01	0.01	0.02	0.01	0.01
1959	-0.01	0.01	0.17	-0.01	0.01	0.20	0.03	0.01	0.00	0.03	0.01	0.00
1960	-0.02	0.01	0.02	-0.02	0.01	0.07	0.04	0.01	0.00	0.04	0.01	0.00
1961	-0.02	0.01	0.06	-0.02	0.01	0.10	0.06	0.01	0.00	0.06	0.01	0.00
1962	-0.04	0.01	0.00	-0.04	0.01	0.00	0.05	0.01	0.00	0.05	0.01	0.00
1963	-0.04	0.01	0.00	-0.03	0.01	0.01	0.06	0.01	0.00	0.06	0.01	0.00
1964	-0.04	0.01	0.00	-0.03	0.01	0.00	0.07	0.01	0.00	0.07	0.01	0.00
1965	-0.05	0.01	0.00	-0.05	0.01	0.00	0.07	0.01	0.00	0.07	0.01	0.00
1966	-0.07	0.01	0.00	-0.07	0.01	0.00	0.05	0.01	0.00	0.05	0.01	0.00
1967	-0.06	0.01	0.00	-0.05	0.01	0.00	0.06	0.01	0.00	0.06	0.01	0.00
1968	-0.07	0.01	0.00	-0.06	0.01	0.00	0.07	0.01	0.00	0.07	0.01	0.00
1969	-0.08	0.01	0.00	-0.08	0.01	0.00	0.06	0.01	0.00	0.06	0.01	0.00
1970	-0.09	0.01	0.00	-0.08	0.01	0.00	0.06	0.01	0.00	0.06	0.01	0.00
1971	-0.09	0.01	0.00	-0.09	0.01	0.00	0.07	0.01	0.00	0.07	0.01	0.00
1972	-0.08	0.01	0.00	-0.08	0.01	0.00	0.08	0.01	0.00	0.08	0.01	0.00
1973	-0.07	0.01	0.00	-0.06	0.01	0.00	0.09	0.01	0.00	0.09	0.01	0.00
1974	-0.07	0.01	0.00	-0.06	0.01	0.00	0.10	0.01	0.00	0.10	0.01	0.00
1975	-0.08	0.01	0.00	-0.07	0.01	0.00	0.09	0.01	0.00	0.09	0.01	0.00

Continues on next page

Mother tongue (Finnish)	Ref.			Ref.			Ref.			Ref.		
Swedish	0.08	0.01	0.00	0.01	0.01	0.13	0.01	0.01	0.38	0.01	0.01	0.38
Other	0.07	0.06	0.27	0.16	0.07	0.02	0.12	0.07	0.07	0.11	0.07	0.08
Educational level (Primary)	Ref.			Ref.			Ref.			Ref.		
Secondary	0.15	0.00	0.00	0.13	0.00	0.00	0.00	0.01	0.95	0.00	0.01	0.91
Tertiary, short-cycle	0.28	0.01	0.00	0.29	0.01	0.00	0.00	0.01	0.76	0.00	0.01	0.81
Tertiary, bachelor	0.33	0.01	0.00	0.34	0.01	0.00	0.01	0.01	0.15	0.01	0.01	0.14
Tertiary, master or higher	0.39	0.01	0.00	0.48	0.01	0.00	0.05	0.01	0.00	0.05	0.01	0.00
Municipality of residence	No			Yes			Yes			Yes		
Marital status (Married)							Ref.			Ref.		
Not married							-1.55	0.00	0.00	-1.55	0.00	0.00
Divorced							-0.19	0.01	0.00	-0.19	0.01	0.00
Widow(er)							-0.43	0.03	0.00	-0.43	0.03	0.00
Change of religious denomination (No)										Ref.		
Yes										-0.03	0.01	0.00

Educational level, Mother tounge, Muncipality of residence, and Marital status refers to the situation at age 45.
Change of religious denomination refers to the situation at age 45 versus at age 15.
Average marginal effects of Municipality of residence are not displayed because of the large number of parameters (457).

Table A4. Average marginal effects (with standard errors and p-values) of the control variables referred to in Table 3

	Model 2			Model 3			Model 4			Model 5		
	dy/dx	S.E.	P>|z|	dy/dx	S.E.	P>|z|	dy/dx	S.E.	P>|z|	dy/dx	S.E.	P>|z|
WOMEN												
Birth cohort (1956)	Ref.			Ref.			Ref.			Ref.		
1957	0.03	0.01	0.01	0.03	0.01	0.01	0.04	0.01	0.00	0.04	0.01	0.00
1958	0.05	0.01	0.00	0.05	0.01	0.00	0.06	0.01	0.00	0.06	0.01	0.00
1959	0.05	0.01	0.00	0.05	0.01	0.00	0.07	0.01	0.00	0.07	0.01	0.00
1960	0.07	0.01	0.00	0.07	0.01	0.00	0.09	0.01	0.00	0.09	0.01	0.00
1961	0.07	0.01	0.00	0.07	0.01	0.00	0.10	0.01	0.00	0.10	0.01	0.00
1962	0.08	0.01	0.00	0.08	0.01	0.00	0.12	0.01	0.00	0.12	0.01	0.00
1963	0.08	0.01	0.00	0.09	0.01	0.00	0.13	0.01	0.00	0.13	0.01	0.00
1964	0.07	0.01	0.00	0.08	0.01	0.00	0.12	0.01	0.00	0.12	0.01	0.00
1965	0.09	0.01	0.00	0.10	0.01	0.00	0.15	0.01	0.00	0.15	0.01	0.00
1966	0.07	0.01	0.00	0.09	0.01	0.00	0.14	0.01	0.00	0.14	0.01	0.00
1967	0.06	0.01	0.00	0.08	0.01	0.00	0.13	0.01	0.00	0.13	0.01	0.00
1968	0.08	0.01	0.00	0.10	0.01	0.00	0.16	0.01	0.00	0.16	0.01	0.00
1969	0.08	0.01	0.00	0.09	0.01	0.00	0.15	0.01	0.00	0.15	0.01	0.00
1970	0.08	0.01	0.00	0.09	0.01	0.00	0.15	0.01	0.00	0.15	0.01	0.00
1971	0.09	0.01	0.00	0.11	0.01	0.00	0.17	0.01	0.00	0.17	0.01	0.00
1972	0.12	0.01	0.00	0.13	0.01	0.00	0.19	0.01	0.00	0.19	0.01	0.00
1973	0.10	0.01	0.00	0.11	0.01	0.00	0.17	0.01	0.00	0.17	0.01	0.00
1974	0.09	0.01	0.00	0.10	0.01	0.00	0.17	0.01	0.00	0.17	0.01	0.00
1975	0.10	0.01	0.00	0.11	0.01	0.00	0.18	0.01	0.00	0.18	0.01	0.00
Mother tongue (Finnish)	Ref.			Ref.			Ref.			Ref.		
Swedish	0.04	0.01	0.00	0.04	0.01	0.00	0.05	0.01	0.00	0.05	0.01	0.00
Other	-0.12	0.08	0.16	-0.06	0.09	0.49	-0.05	0.09	0.55	-0.06	0.09	0.46
Educational level (Primary)	Ref.			Ref.			Ref.			Ref.		
Secondary	0.08	0.01	0.00	0.04	0.01	0.00	0.01	0.01	0.29	0.01	0.01	0.27
Tertiary, short-cycle	-0.03	0.01	0.00	-0.04	0.01	0.00	-0.10	0.01	0.00	-0.10	0.01	0.00
Tertiary, bachelor	0.04	0.01	0.00	0.02	0.01	0.02	-0.05	0.01	0.00	-0.05	0.01	0.00
Tertiary, master or higher	-0.01	0.01	0.45	0.02	0.01	0.02	-0.08	0.01	0.00	-0.08	0.01	0.00
Municipality of residence	No			Yes			Yes			Yes		
Marital status (Married)							Ref.			Ref.		
Not married							-0.63	0.01	0.00	-0.63	0.01	0.00
Divorced							-0.23	0.01	0.00	-0.23	0.01	0.00
Widow(er)							-0.25	0.03	0.00	-0.25	0.03	0.00
Change of religious denomination (No)										Ref.		
Yes										-0.08	0.01	0.00
MEN												
Birth cohort (1956)	Ref.			Ref.			Ref.			Ref.		
1957	0.02	0.01	0.17	0.02	0.01	0.14	0.03	0.01	0.03	0.03	0.01	0.03
1958	0.01	0.01	0.38	0.01	0.01	0.36	0.02	0.01	0.06	0.02	0.01	0.06
1959	0.02	0.01	0.17	0.02	0.01	0.14	0.04	0.01	0.00	0.04	0.01	0.00
1960	0.01	0.01	0.29	0.02	0.01	0.12	0.04	0.01	0.00	0.04	0.01	0.00
1961	0.02	0.01	0.14	0.03	0.01	0.06	0.06	0.01	0.00	0.06	0.01	0.00
1962	0.01	0.01	0.56	0.01	0.01	0.30	0.05	0.01	0.00	0.05	0.01	0.00
1963	0.01	0.01	0.32	0.03	0.01	0.03	0.06	0.01	0.00	0.06	0.01	0.00
1964	0.01	0.01	0.33	0.03	0.01	0.02	0.07	0.01	0.00	0.07	0.01	0.00
1965	0.02	0.01	0.19	0.03	0.01	0.02	0.07	0.01	0.00	0.07	0.01	0.00
1966	0.00	0.01	1.00	0.02	0.01	0.26	0.06	0.01	0.00	0.06	0.01	0.00
1967	0.01	0.01	0.28	0.03	0.01	0.02	0.07	0.01	0.00	0.08	0.01	0.00
1968	0.00	0.01	0.81	0.02	0.01	0.13	0.07	0.01	0.00	0.07	0.01	0.00
1969	0.00	0.01	0.91	0.01	0.01	0.31	0.07	0.01	0.00	0.07	0.01	0.00
1970	0.00	0.01	0.93	0.01	0.01	0.39	0.06	0.01	0.00	0.06	0.01	0.00
1971	0.01	0.01	0.52	0.03	0.01	0.08	0.08	0.01	0.00	0.08	0.01	0.00
1972	0.01	0.01	0.38	0.03	0.01	0.06	0.08	0.01	0.00	0.08	0.01	0.00
1973	0.03	0.01	0.07	0.04	0.01	0.01	0.10	0.01	0.00	0.10	0.01	0.00
1974	0.01	0.01	0.47	0.02	0.01	0.15	0.09	0.01	0.00	0.09	0.01	0.00
1975	0.00	0.01	0.93	0.02	0.01	0.27	0.08	0.01	0.00	0.08	0.01	0.00

Continues on next page

Mother tongue (Finnish)	Ref.			Ref.			Ref.			Ref.		
Swedish	0.01	0.01	0.42	-0.01	0.01	0.52	-0.01	0.01	0.38	-0.01	0.01	0.37
Other	0.02	0.08	0.82	0.11	0.09	0.23	0.11	0.09	0.22	0.09	0.09	0.29
Educational level (Primary)	Ref.			Ref.			Ref.			Ref.		
Secondary	0.05	0.01	0.00	0.03	0.01	0.00	-0.01	0.01	0.39	-0.01	0.01	0.43
Tertiary, short-cycle	0.07	0.01	0.00	0.08	0.01	0.00	0.00	0.01	0.73	0.00	0.01	0.79
Tertiary, bachelor	0.10	0.01	0.00	0.12	0.01	0.00	0.02	0.01	0.03	0.02	0.01	0.03
Tertiary, master or higher	0.15	0.01	0.00	0.23	0.01	0.00	0.09	0.01	0.00	0.09	0.01	0.00
Municipality of residence	No			Yes			Yes			Yes		
Marital status (Married)							Ref.			Ref.		
Not married							-0.63	0.01	0.00	-0.63	0.01	0.00
Divorced							-0.26	0.01	0.00	-0.26	0.01	0.00
Widow(er)							-0.54	0.07	0.00	-0.54	0.07	0.00
Change of religious denomination (No)										Ref.		
Yes										-0.07	0.01	0.00

Educational level, Mother tounge, Muncipality of residence, and Marital status refers to the situation at age 45.
Change of religious denomination refers to the situation at age 45 versus at age 15.
Average marginal effects of Municipality of residence are not displayed because of the large number of parameters (457).

Table A5. Average marginal effects (with standard errors and p-values) of the control variables referred to in Table A1

	Model 2			Model 3			Model 4		
	dy/dx	S.E.	P>|z|	dy/dx	S.E.	P>|z|	dy/dx	S.E.	P>|z|
WOMEN									
Birth cohort (1956)	Ref.			Ref.			Ref.		
1957	0.03	0.01	0.01	0.03	0.01	0.01	0.03	0.01	0.00
1958	0.04	0.01	0.00	0.04	0.01	0.00	0.06	0.01	0.00
1959	0.04	0.01	0.00	0.04	0.01	0.00	0.07	0.01	0.00
1960	0.05	0.01	0.00	0.06	0.01	0.00	0.10	0.01	0.00
1961	0.04	0.01	0.00	0.05	0.01	0.00	0.10	0.01	0.00
1962	0.04	0.01	0.00	0.04	0.01	0.00	0.12	0.01	0.00
1963	0.04	0.01	0.00	0.06	0.01	0.00	0.14	0.01	0.00
1964	0.03	0.01	0.01	0.05	0.01	0.00	0.14	0.01	0.00
1965	0.03	0.01	0.00	0.05	0.01	0.00	0.16	0.01	0.00
1966	0.02	0.01	0.06	0.04	0.01	0.00	0.15	0.01	0.00
1967	0.01	0.01	0.40	0.04	0.01	0.00	0.15	0.01	0.00
1968	0.03	0.01	0.00	0.06	0.01	0.00	0.17	0.01	0.00
1969	0.03	0.01	0.00	0.05	0.01	0.00	0.18	0.01	0.00
1970	0.01	0.01	0.29	0.03	0.01	0.01	0.16	0.01	0.00
1971	0.02	0.01	0.03	0.05	0.01	0.00	0.19	0.01	0.00
1972	0.05	0.01	0.00	0.06	0.01	0.00	0.21	0.01	0.00
1973	0.04	0.01	0.00	0.06	0.01	0.00	0.19	0.01	0.00
1974	0.03	0.01	0.00	0.06	0.01	0.00	0.20	0.01	0.00
1975	0.05	0.01	0.00	0.07	0.01	0.00	0.22	0.01	0.00
Mother tongue (Finnish)	Ref.			Ref.			Ref.		
Swedish	0.07	0.01	0.00	0.05	0.01	0.00	0.08	0.01	0.00
Other	-0.11	0.06	0.09	-0.02	0.07	0.73	-0.01	0.07	0.93
Educational level (Primary)	Ref.			Ref.			Ref.		
Secondary	0.14	0.01	0.00	0.08	0.01	0.00	-0.01	0.01	0.18
Tertiary, short-cycle	-0.01	0.01	0.07	-0.04	0.01	0.00	-0.19	0.01	0.00
Tertiary, bachelor	0.05	0.01	0.00	0.04	0.01	0.00	-0.14	0.01	0.00
Tertiary, master or higher	-0.05	0.01	0.00	0.01	0.01	0.23	-0.21	0.01	0.00
Municipality of residence	No			Yes			Yes		
Marital status (Married)							Ref.		
Not married							-1.32	0.00	0.00
Divorced							-0.13	0.01	0.00
Widow(er)							-0.17	0.02	0.00
Change of religious denomination (No)									
Yes									
Birth cohort (1956)	Ref.			Ref.			Ref.		
1957	0.02	0.01	0.10	0.02	0.01	0.06	0.03	0.01	0.00
1958	-0.01	0.01	0.60	0.00	0.01	0.70	0.02	0.01	0.01
1959	-0.01	0.01	0.15	-0.01	0.01	0.18	0.03	0.01	0.00
1960	-0.02	0.01	0.02	-0.02	0.01	0.06	0.04	0.01	0.00
1961	-0.02	0.01	0.05	-0.02	0.01	0.08	0.06	0.01	0.00
1962	-0.04	0.01	0.00	-0.04	0.01	0.00	0.05	0.01	0.00
1963	-0.04	0.01	0.00	-0.03	0.01	0.01	0.06	0.01	0.00
1964	-0.04	0.01	0.00	-0.03	0.01	0.00	0.07	0.01	0.00
1965	-0.05	0.01	0.00	-0.05	0.01	0.00	0.07	0.01	0.00
1966	-0.07	0.01	0.00	-0.07	0.01	0.00	0.05	0.01	0.00
1967	-0.07	0.01	0.00	-0.05	0.01	0.00	0.06	0.01	0.00
1968	-0.07	0.01	0.00	-0.06	0.01	0.00	0.07	0.01	0.00
1969	-0.08	0.01	0.00	-0.08	0.01	0.00	0.06	0.01	0.00
1970	-0.09	0.01	0.00	-0.08	0.01	0.00	0.06	0.01	0.00
1971	-0.10	0.01	0.00	-0.09	0.01	0.00	0.07	0.01	0.00
1972	-0.08	0.01	0.00	-0.08	0.01	0.00	0.08	0.01	0.00
1973	-0.07	0.01	0.00	-0.06	0.01	0.00	0.09	0.01	0.00
1974	-0.07	0.01	0.00	-0.06	0.01	0.00	0.10	0.01	0.00
1975	-0.08	0.01	0.00	-0.07	0.01	0.00	0.09	0.01	0.00

Continues on next page

Mother tongue (Finnish)	Ref.			Ref.			Ref.		
Swedish	0.08	0.01	0.00	0.02	0.01	0.11	0.01	0.01	0.37
Other	0.07	0.06	0.29	0.15	0.07	0.03	0.12	0.07	0.08
Educational level (Primary)	Ref.			Ref.			Ref.		
Secondary	0.15	0.00	0.00	0.13	0.00	0.00	0.00	0.01	0.81
Tertiary, short-cycle	0.29	0.01	0.00	0.29	0.01	0.00	0.00	0.01	0.95
Tertiary, bachelor	0.33	0.01	0.00	0.34	0.01	0.00	0.01	0.01	0.09
Tertiary, master or higher	0.39	0.01	0.00	0.48	0.01	0.00	0.06	0.01	0.00
Municipality of residence	No			Yes			Yes		
Marital status (Married)							Ref.		
Not married							-1.55	0.00	0.00
Divorced							-0.19	0.01	0.00
Widow(er)							-0.43	0.03	0.00

Educational level, Mother tounge, Muncipality of residence, and Marital status refers to the situation [illegible]
Average marginal effects of Municipality of residence are not displayed because of the large number of parameters (457).
